# Mechanisms and Evolutionary Patterns of Mammalian and Avian Dosage Compensation

**DOI:** 10.1371/journal.pbio.1001328

**Published:** 2012-05-15

**Authors:** Philippe Julien, David Brawand, Magali Soumillon, Anamaria Necsulea, Angélica Liechti, Frédéric Schütz, Tasman Daish, Frank Grützner, Henrik Kaessmann

**Affiliations:** 1 Center for Integrative Genomics, University of Lausanne, Switzerland; 2 Swiss Institute of Bioinformatics, Lausanne, Switzerland; 3 The Robinson Institute, School of Molecular and Biomedical Science, University of Adelaide, Australia; University of Edinburgh, United Kingdom

## Abstract

As a result of sex chromosome differentiation from ancestral autosomes, male mammalian cells only contain one X chromosome. It has long been hypothesized that X-linked gene expression levels have become doubled in males to restore the original transcriptional output, and that the resulting X overexpression in females then drove the evolution of X inactivation (XCI). However, this model has never been directly tested and patterns and mechanisms of dosage compensation across different mammals and birds generally remain little understood. Here we trace the evolution of dosage compensation using extensive transcriptome data from males and females representing all major mammalian lineages and birds. Our analyses suggest that the X has become globally upregulated in marsupials, whereas we do not detect a global upregulation of this chromosome in placental mammals. However, we find that a subset of autosomal genes interacting with X-linked genes have become downregulated in placentals upon the emergence of sex chromosomes. Thus, different driving forces may underlie the evolution of XCI and the highly efficient equilibration of X expression levels between the sexes observed for both of these lineages. In the egg-laying monotremes and birds, which have partially homologous sex chromosome systems, partial upregulation of the X (Z in birds) evolved but is largely restricted to the heterogametic sex, which provides an explanation for the partially sex-biased X (Z) expression and lack of global inactivation mechanisms in these lineages. Our findings suggest that dosage reductions imposed by sex chromosome differentiation events in amniotes were resolved in strikingly different ways.

## Abstract 2

A large-scale comparative gene expression study reveals the different ways in which the chromosome-wide gene dosage reductions resulting from sex chromosome differentiation events were compensated during mammalian and avian evolution.

## Author Summary

Mammalian sex chromosomes (the X and Y) evolved from an ordinary pair of ancestral somatic chromosomes (the proto-sex chromosomes). The process that led to emergence of distinct sex chromosomes involved the degeneration of the Y chromosome, leaving males with only one copy of most proto-sex chromosomal genes on their single X chromosome. It has remained unclear whether mechanisms evolved that compensate for this dosage reduction. Here we trace the evolution of sex chromosomal expression levels in all major mammalian lineages and in birds. We find that the X has become globally upregulated in response to the dosage reduction in marsupials, whereas in placental mammals, genes resident on autosomal (non-sex) chromosomes that interact with X-linked genes have instead become downregulated. These mechanisms restore ancestral gene expression balances and also presumably drove the evolution of secondary compensation mechanisms (i.e., female X-inactivation) in these mammalian lineages. In egg-laying mammals and birds, sex chromosomes have become partially upregulated specifically in the heterogametic sex, i.e., in male monotremes (which are XY) and female birds (which are WZ). This probably explains why the evolution of inactivation mechanisms in the homogametic sexes in these lineages (XX and ZZ, respectively) was not necessary. Our findings suggest that gene dosage alterations associated with the emergence of sex chromosome systems can be compensated in various different ways.

## Introduction

**Figure 1:**
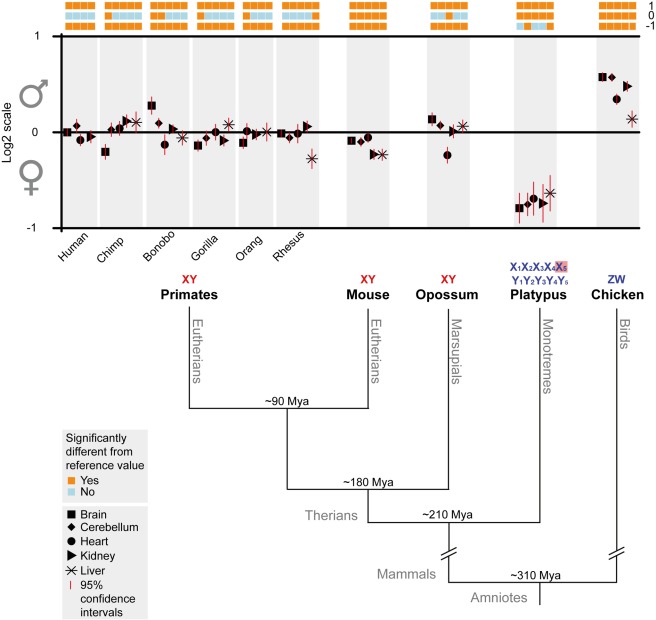
Median male versus female expression levels of mammalian X-linked and avian Z-linked genes in five somatic tissues. Top: Median male to female (M∶F) gene expression level ratios and 95% confidence intervals for five somatic tissues derived from nine mammals and one bird. M∶F ratio calculations are based on genes expressed in both sexes (RPKM>0). Values are plotted on a log_2_ scale to allow for linear and symmetrical patterns (e.g., same distances for two-fold higher expression levels in males or females, respectively). Statistically significant deviations of M∶F ratios from key reference values (orange/blue boxes): 0.5 (log_2_ ratio of −1); 1 (log_2_ ratio of 0); and 2 (log_2_ ratio of 1), as assessed by one-sample Wilcoxon signed rank tests (Bonferroni corrected *p*<0.05). Numbers of X (X_5_, Z) genes considered in the analysis are: 664 (human), 520 (chimp), 657 (gorilla), 606 (orang), 731 (macaque), 750 (mouse), 442 (opossum), 137 (platypus), 733 (chicken). Bottom: Schematic tree illustrating the phylogenetic relationships and sex chromosomes (homologous therian XY chromosomes in red; [partially] homologous platypus and bird sex chromosomes in blue) of the amniote lineages for which male and female expression was compared. Specifically, male and female expression values were compared for the therian X, platypus X_5_ (highlighted in pink), and chicken Z chromosome. Approximate divergence time estimates (million years ago [Mya]) are based on previous studies [52]–[55]. Note that the expression ratios shown for eutherians are based on protein-coding genes from the entire X chromosome, that is, the ancestral part of the eutherian X (the so-called XCR) [31], as well as the region that became X-linked during early eutherian evolution (termed XAR) [31]. Expression ratios for the XCR only are shown in Figure S1.

In mammals and birds, sex is determined by pairs of heteromorphic sex chromosomes that differentiated from ancestral autosomes [1]. All mammals evolved sex chromosomes with male heterogamety (XY system), but different sets of ancestral autosomes evolved into sex chromosomes in therian (placental/marsupial) and monotreme mammals (Figure 1). Thus, placental mammals (eutherians) and marsupials share the same X and Y, whereas the multiple X and Y chromosomes of the egg-laying monotremes are distinct and partially homologous to the sex chromosomes of birds [2]–[4], where females are heterogametic (ZW system).

The process of sex chromosome differentiation in mammals and birds involved the degeneration of the sex-specific chromosome [5]–[7]. The prevailing theory, originally put forward by Susumu Ohno in 1967 [1], posits that this dosage reduction in the heterogametic sex led to the evolution of dosage compensation mechanisms in mammals [8]–[10]. Specifically, to compensate for the two-fold reduction of the transcriptional output from the remaining single X in males, X-linked genes are thought to have evolved two-fold higher expression levels, which restored ancestral transcript levels of the X in males and thus also maintained the balance between X-linked and autosomal gene expression in this sex. The resulting overabundance of X transcripts resulting from the combined activity of the two upregulated X chromosomes in females was then compensated by the inactivation of one of the X chromosomes (XCI).

Several previous studies sought to assess the extent of X upregulation in eutherians on the basis of comparisons of current expression levels between present-day X-linked and autosomal genes. Initial analyses of human and mouse microarray data suggested an approximately two-fold upregulation of the single active X in eutherians, on the basis of the observation that the extant X and autosomes have overall similar transcriptional outputs [11],[12]. However, a subsequent study by Xiong et al., based on RNA sequencing (RNA-seq) data, found the X to only have approximately half of the transcriptional output of autosomes, thus questioning the occurrence of X upregulation [13].

Recently, however, three other RNA-seq–based studies [14]–[16] and a reanalysis of microarray data [17] restored the original claim of X upregulation, suggesting that the low expression levels of the X inferred in the Xiong et al. study were due to the inclusion of genes with little or no expression [14]. Previous studies have thus reached inconsistent conclusions regarding the occurrence of X upregulation. However, it is important to point out that the analyses in all of these previous studies were indirect. They implicitly assumed that genes on the ancestral proto-X chromosomes (i.e., the autosomal progenitors of the X) were expressed at the same level as genes on ancestral autosomes before sex chromosome differentiation and that, therefore, similar expression levels between the single active present-day X and autosomes are indicative of X upregulation. Furthermore, the assessment of X upregulation patterns (i.e., the extent of restoration of ancestral expression levels on the X after Y decay) is only relevant for genes already present on the proto-X, but only Xiong et al. included analyses specifically for such ancestral X-linked genes [13]. Thus, to more directly assess the occurrence of X upregulation as a response to sex chromosome differentiation (and hence to test Ohno's original hypothesis [1]), current X expression levels need to be compared to ancestral X expression levels (relative to the respective autosomal backgrounds), for genes that were already present on the proto-X [18].

It is currently unclear whether marsupials evolved efficient and global dosage compensation mechanisms in response to Y chromosome decay, because of the limited number of genes for which expression patterns have been assessed. However, previous studies showed that the X stemming from the father is, at least to some extent, inactivated in the soma of female marsupials through specific epigenetic chromatin modifications [19],[20]. Transcriptional silencing of the paternal X in marsupials was reported to be quite leaky and unstable [21]–[23], although a recent study revealed efficient XCI for at least some genes [24]. Notably, the apparently paternally imprinted XCI observed in extant marsupials was hypothesized to reflect properties of an ancestral therian mechanism, which was then replaced by the random and potentially more efficient XCI mechanism in eutherians that is dependent on the *Xist* gene, which is absent in marsupials [25].

Even less is known about potential patterns of dosage compensation in the egg-laying monotremes, the third major mammalian lineage. An initial study of individual genes in platypus fibroblast cell lines indicated that only some X-linked genes might be dosage compensated and only to a certain degree because of variable patterns of XCI among cells [26]. Also, a recent immunofluorescence analysis of epigenetic modifications of the platypus X chromosomes provided no evidence for chromosome-wide XCI in monotremes [20]. Notably, it was suggested that the evolution of global dosage compensation mechanisms may not necessarily accompany the differentiation of sex chromosomes in amniotes (i.e., mammals, birds, reptiles), because birds were reported to lack chromosome-wide (Z) dosage compensation, on the basis of the observation of significantly elevated Z expression levels in males, the homogametic sex in birds [27]–[29].

Overall, previous observations point to fundamental differences between lineages with respect to patterns of dosage compensation and the associated selective forces. To assess in detail the patterns, mechanisms, and evolutionary driving forces of dosage compensation in mammals and birds, we exploited an extensive set of transcriptome data that we recently generated using high-throughput RNA-seq for a collection of six major organs (cerebellum, cortex, heart, kidney, liver, testis) derived from males and females from ten species that represent all major mammalian lineages and birds (Methods) (Figure 1) [30]. In addition, we produced complementary RNA-seq data (fibroblasts, ovary) for specific analyses of platypus dosage compensation patterns (Methods).

### Male Versus Female Expression Levels in Eutherians

**Figure 2:**
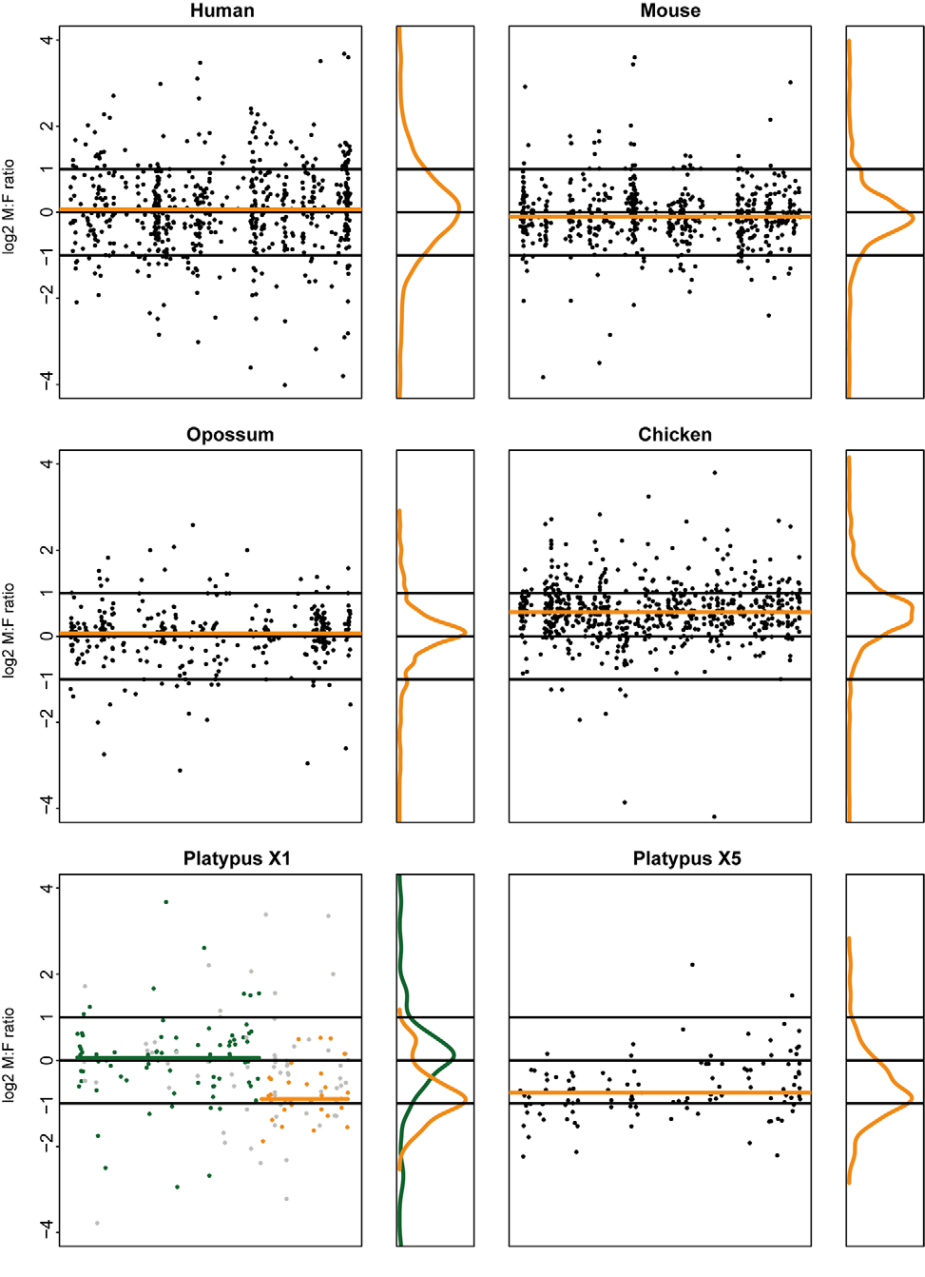
Male versus female expression levels of individual sex chromosomal genes in cerebellum from representative amniotes. Male to female (M∶F) gene expression ratios are plotted for individual genes (expressed in both sexes) on the human, mouse, and opossum X, platypus X_1_ and X_5_, and chicken Z chromosome for a representative tissue (cerebellum). Values are plotted on a log_2_ scale to allow for linear and symmetrical patterns (e.g., 0.5 [log_2_ ratio of −1]; 1 [log_2_ ratio of 0]; and 2 [log_2_ ratio of 1]). Median M∶F ratios for human, mouse, and opossum X, platypus X_5_ and X_1_ non-pseudoautosomal region, and chicken Z are indicated by orange lines. The distribution of individual M∶F ratios (orange vertical plots) is shown to the right of each main plot. Expression ratios of individual genes on the platypus X_1_ chromosome are indicated by green circles (pseudoautosomal genes with 1∶1 orthologs on chicken Chromosomes Z, 3, or 13; see [3] for details), orange circles (non-pseudoautosomal/sex-linked genes with 1∶1 orthologs on chicken Chromosome 12 [3]), or grey circles (pseudoautosomal or non-pseudoautosomal genes without clear 1∶1 chicken orthologs). Note that the respective medians for the platypus X_1_ were calculated on the basis of genes with chicken 1∶1 orthologs, although the other genes in these regions show very similar patterns. See Table S3 for platypus X_1_ M∶F ratios for all six organs.

**Figure 3:**
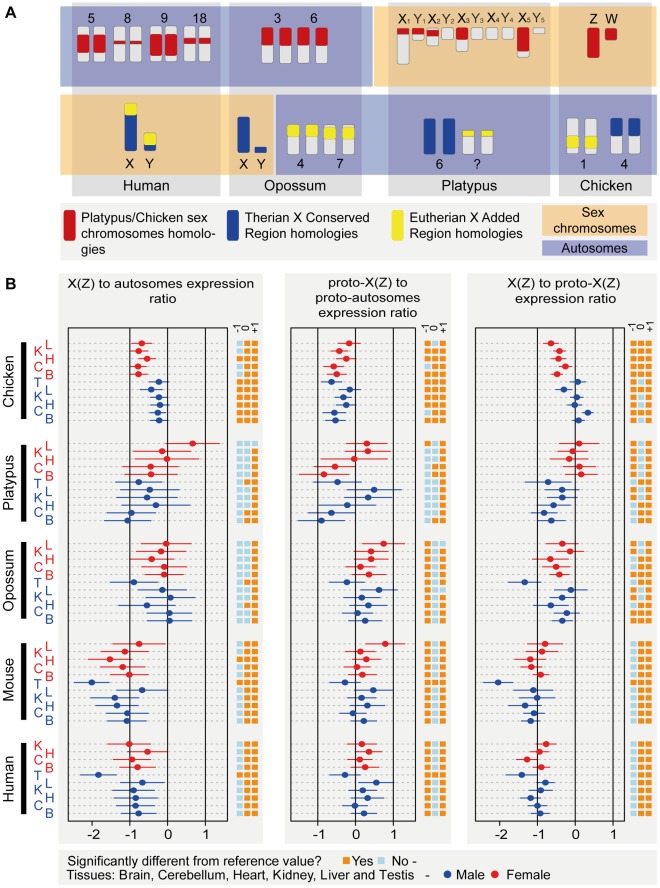
Sex chromosome homology relationships and current and inferred ancestral expression levels of genes on the mammalian (proto) X or avian Z chromosomes. (A) Sex chromosomes in the different mammals and birds and their corresponding homologous autosomal counterparts in species with non-homologous sex chromosome systems. (B) Left: median X (Z) to autosome expression level ratios (and 95% confidence intervals) of expressed genes on the current sex chromosomes in five representative amniotes that have 1∶1 orthologs in all studied species (see Figure S3 for plots with all ten species). Middle: median X (Z) to autosome ratios of expressed genes on “proto-sex chromosomes,” as inferred from autosomal one-to-one orthologous genes from species with non-homologous sex chromosomes (see (A), main text, and Methods for details). Right: median current to ancestral X (Z)-linked gene expression ratios for genes expressed both on the current X and proto-X (normalized by expression levels of autosomal genes, respectively). Note that values are plotted on a log_2_ scale to allow for linear and symmetrical patterns. Numbers of X (X_5_, Z) conserved genes (i.e., genes with clear 1∶1 orthologs across the ten species) considered in these analyses are: 157 (human), 156 (chimp), 158 (gorilla), 156 (orang), 155 (macaque), 153 (mouse), 91 (opossum), 56 (platypus), 296 (chicken). See Figure S3 for similar plot containing data for all ten species. See Methods for details regarding the calculation of the different ratios. Statistically significant deviations from the reference values (0.5 [log_2_ ratio of −1]; 1 [log_2_ ratio of 0]; and 2 [log_2_ ratio of 1]), as assessed by one-sample Wilcoxon signed rank tests (Benjamini-Hochberg corrected *p*<0.05) are indicated to the right of each plot (oranges/blue boxes).

To assess patterns of dosage compensation in eutherians, we first contrasted male and female expression levels by computing global male to female (M∶F) gene expression ratios for expressed genes in a given tissue in both sexes (Methods). Our analyses of the data for the five somatic tissues from the seven eutherian species (humans and the other four great apes, macaque, and mouse) show that median expression levels of X-linked genes are generally very similar and statistically indistinguishable between males and females in 22 out of 33 comparisons (Figures 1 and 2, for chromosome-wide pattern of specific human and mouse examples). Notably, in nine of the 11 sex-biased cases, expression was significantly higher in females (i.e., M∶F ratios<1; Bonferroni corrected *p*<0.05, one-sample Wilcoxon signed rank test), which may be indicative of leaky expression of some genes from an incompletely inactivated female X [8]. In agreement with this notion, the number of female-biased cases is reduced to three when the analysis is restricted to the original part of the X that is shared with marsupials (the so-called X-conserved region [XCR]) (Figure 3A) [31]; that is, when the eutherian-specific region of the X (the so-called X-added region [XAR]) (Figure 3A) [31], known to contain the majority of genes escaping inactivation [8], is excluded from the analysis (Figure S1). In addition, general inter-individual expression differences of X-linked genes (e.g., due to environmental effects during sampling) may account for the slightly sex-biased patterns observed, in particular with respect to the two bonobo tissues that display male-biased expression. Overall, our observations are consistent with previous studies in humans and mouse [8]. They thus support the notion that all eutherians evolved X dosage compensation mechanisms that result in very similar expression levels between the two sexes.

### Current X to Autosome Expression Comparisons in Eutherians

However, the original driving force for the evolution of dosage compensation mechanisms ought to have been the selective pressure to upregulate the single X in males upon Y chromosome decay (see above) [1],[9]. Thus, to understand the mechanisms and driving forces of dosage compensation, one needs to assess whether, or to what extent, ancestral expression levels have been restored through upregulation of the X.

Previous studies sought to assess the extent of X upregulation on the basis of comparisons of current expression levels between genes on the extant X chromosome and autosomes [11],[13],[14],[16],[32]. To place our study in the context of these studies, we first assessed current X-to-autosome (X∶AA) expression ratios for somatic tissues in the seven eutherian species on the basis of median expression levels of expressed genes on the two types of chromosomes. X∶AA ratios are always significantly smaller than 1 but often significantly larger than 0.5 (Figure S2). The median X∶AA value is 0.71 in primates and 0.5 in mouse (Table S1). These values are very similar to the values reported by Deng et al. (human, 0.72; mouse, 0.57) [14]. However, they are overall higher than what was reported in another RNA-seq–based study by Xiong et al. (human, 0.47; mouse, 0.2) [13], probably due to the inclusion of non-expressed genes in that study [14]. Generally, it was suggested that it is important to assess entire distributions of expression levels, given that median estimates might be influenced by lowly expressed genes, or by genes lacking expression in a given tissue [14]. Our analyses reveal significant (*p*<0.05; Benjamini-Hochberg corrected Komolgorov-Smirnov test) shifts of X expression level distributions towards lower values compared to autosomal distributions for most but not all tissues (e.g., human brain) in the different species (Figure S3; Table S1), in good agreement with previous results [14]. The fact that X expression level distributions are sometimes only slightly shifted towards lower values, or not significantly shifted at all, were interpreted to provide evidence for the presence of X upregulation mechanisms [14].

### The Evolution of Dosage Compensation in Eutherians

However, as pointed out above (see Introduction), a more direct assessment of X dosage compensation (i.e., the extent of restoration of ancestral expression levels on the X after Y decay) requires comparisons of current X expression levels with ancestral expression levels (relative to the respective autosomal expression levels), for genes that were already present on the proto-X. In this context, it is noteworthy that we observe significant variability in global transcriptional output of chromosomes in the different tissues in all eutherians (Benjamini-Hochberg corrected *p*<0.05, Kruskal-Wallis test), except for one sample (female orang-utan heart). This indicates that chromosomes should not be expected to necessarily have similar overall expression levels.

To be able to more directly assess the extent of upregulation of genes on the eutherian X chromosomes after sex chromosome differentiation, we exploited the fact that the current eutherian X is derived from ancestral autosomes. It therefore has autosomal counterparts in species with non-homologous sex chromosomes, which are informative with respect to proto-sex chromosome expression patterns (Figure 3A). Thus, the ancestral portion of the eutherian X chromosome largely corresponds to Chromosome 6 in platypus and to part of Chromosome 4 in chicken (Figure 3A). The expression levels of these platypus and chicken autosomes, unaffected by sex chromosome-related selective forces, can therefore be used to gauge ancestral therian X expression levels. Specifically, we compared transcript abundance of genes on the eutherian X in males and females to the transcriptional output of 1∶1 orthologous genes on the corresponding autosomes from platypus and chicken, relative to the respective autosomal background expression (Methods). As controls, we performed similar comparisons for corresponding numbers of 1∶1 orthologous genes that are located on autosomes in these species, which shows that expression levels of autosomal genes have overall been highly conserved between species (Figure S4) and validates our approach to infer ancestral expression patterns using outgroup species.

**Figure 4:**
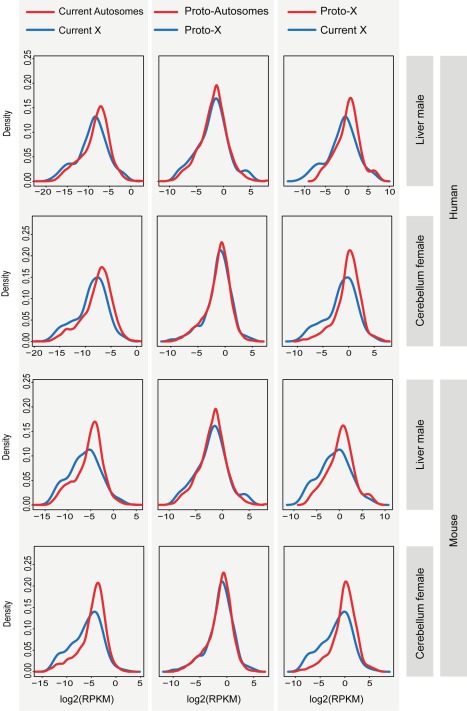
Distributions of current and inferred ancestral expression levels of genes on the eutherian (proto) X chromosomes and autosomes. Distributions of expression levels of (proto) X-linked genes (blue line) and (proto) autosomal genes (red line) are shown for cerebellum and liver from human and mouse. Expression levels in the comparison of the current X and proto-X (right plots) are normalized by the respective autosomal expression levels. Gene expression level distributions for the X are significantly shifted towards lower values compared to those for autosomes (Benjamini-Hochberg *p*<0.05; corrected Komolgorov-Smirnov test). Also, gene expression level distributions for the current X are significantly shifted towards lower values compared to those for the proto-X (Benjamini-Hochberg *p*<0.05; corrected Komolgorov-Smirnov test). The distributions for the proto-X and proto-autosomes are not significantly different from each other (Benjamini-Hochberg *p*>0.05; corrected Komolgorov-Smirnov test). See Table S1 for all tests of differences between X and autosomal expression distributions (all tissues from all species).

Our comparisons of distributions of current expression levels for genes on the X and autosomes with 1∶1 orthologs in the outgroup species reveal significant shifts of X-linked genes towards lower expression values for the vast majority of tissues (71 of 74, 96%) in the different eutherians (Benjamini-Hochberg corrected *p*<0.05, Komolgorov-Smirnov test) (Figure 4; Table S1). By contrast, expression level distributions for the “same” (orthologous) genes are similar between the inferred eutherian proto-X chromosomes (pXX) and ancestral autosomes, which suggests that the therian proto-X chromosomes had a relatively similar transcriptional output as ancestral autosomes (corrected *p*>0.05, Komolgorov-Smirnov test) (Figure 4; Table S1). Consequently, expression values of the current X are significantly lower than those of the proto-X relative to the respective autosomal backgrounds in nearly all (70 of 74, 95%) comparisons of expression level distributions (corrected *p*<0.05, Komolgorov-Smirnov test) (Figure 4; Table S1). Overall, the global expression output from the current single active X relative to autosomes in eutherians is approximately two-fold lower than that inferred for the two proto-X chromosome copies in somatic tissues (Figures 3B and S5), yielding a median current X to proto-X (X∶pXX) expression ratio across somatic tissues of 0.53 for the seven eutherian species (X∶pXX ratio not significantly different from 0.5, corrected *p*>0.05, except for female macaque liver; one-sample Wilcoxon signed rank tests). Notably, analyses restricted to the XCR result in very similar patterns (Figure S6). It is also noteworthy that we obtain very similar results when using more stringent criteria to identify expressed genes (Figure S7; Table S2), or when restricting the X∶pXX calculations to genes with higher expression values for both the X and proto-X (Figure S8; see Text S1 for a discussion of expression cutoffs). Our observations thus indicate that X-linked genes have, generally, not become upregulated in males upon Y decay in eutherians but are expressed at overall similar levels per active allele as their ancestral genes on the proto-X. Notably, X∶pXX ratios are particularly low for the testis (X∶pXX ratios between 0.19 and 0.37; Figures 3B and S5). This observation likely reflects the effect of meiotic sex chromosome inactivation (MSCI) [33], a mechanism that leads to transcriptional silencing of sex chromosomes in male meiotic germ cells and that evolved upon sex chromosome differentiation [2].

### Accumulation of Testis-Specific Genes on the X

**Figure 5:**
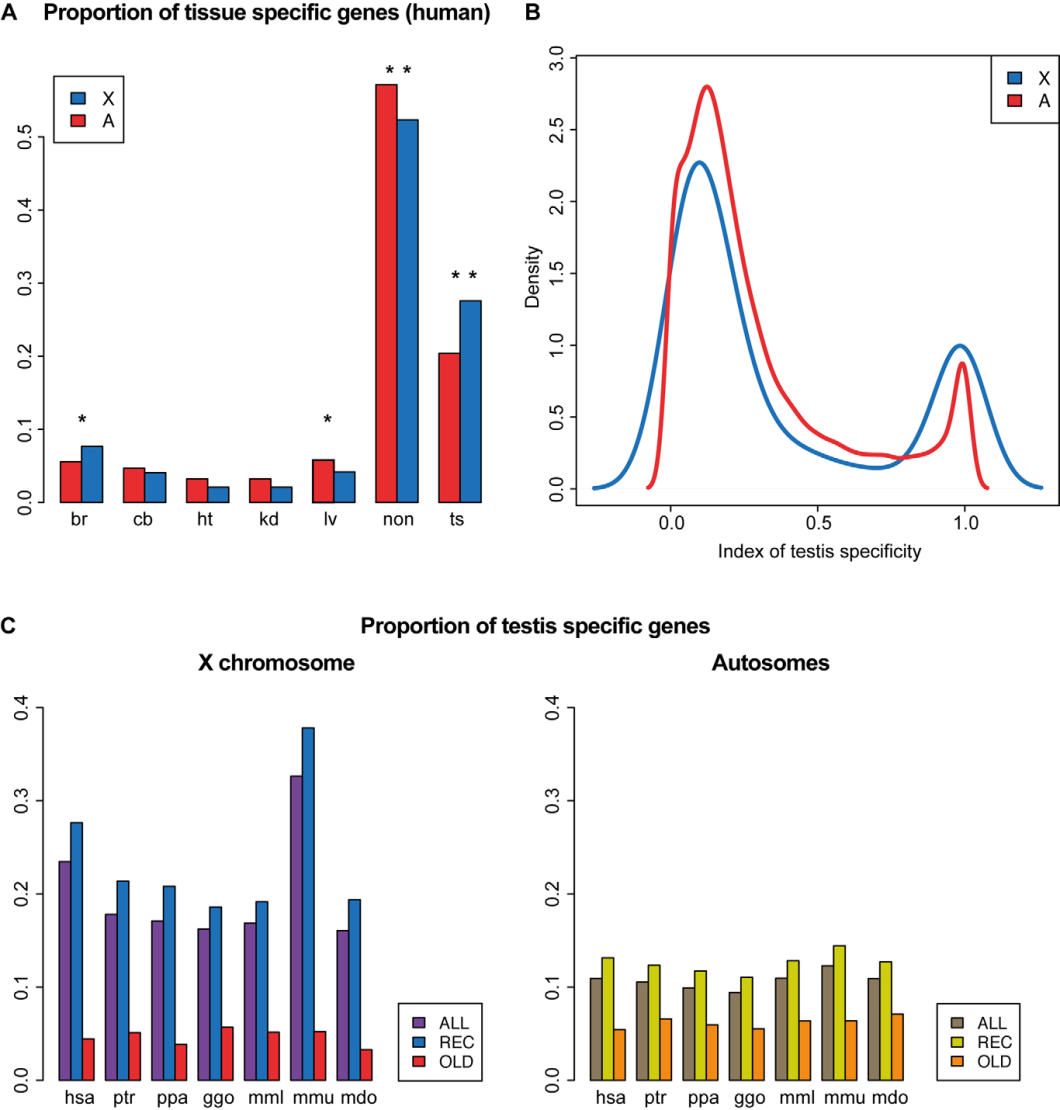
Distributions of tissue-specific genes in the human genome and testis-specific genes therian genomes. (A) Proportions of tissue-specific genes on the human X chromosome and autosomes, respectively: br, brain; cb, cerebellum; ht, heart; kd, kidney; lv, liver; non, no tissue-specificity; and ts, testis. Statistically significant differences as assessed by Fisher exact test are indicated: *two-tailed *p*<0.05; *two-tailed *p*<0.01. (B) Density plots of testis-specificity index for genes on the human X chromosome and autosomes. (C) Proportions of testis-specific genes on therian X chromosomes and autosomes, for all expressed genes (ALL), “recent” genes ([REC], i.e., all genes except 1∶1 orthologs present on both the current chromosomes and ancestral/proto chromosomes), and “old” genes ([OLD], i.e., 1∶1 orthologs present on both the current chromosomes and ancestral/proto chromosomes). Species: hsa, human (*Homo sapiens*); ptr, chimpanzee (*Pan troglodytes*); ppa, bonobo (*Pan paniscus*); ggo, gorilla (*Gorilla gorilla*); mml, macaque (*Macaca mulatta*); mmu, mouse (*Mus musculus*); mdo, opossum (*M. domestica*).

However, the unusual gene content of the X should be taken into account when assessing X upregulation patterns for somatic tissues, as also previously noted [14]. In particular, genes with predominant expression (and functions) in testis seem to be overrepresented on the X [14],[31],[34],[35]. Given that such genes have overall low expression levels and potentially no functions in somatic tissues, inclusion of these genes in comparisons of X and autosomal expression levels may lead to disproportionate reductions of somatic expression level estimates for the X [14]. To address this issue, we first assessed patterns of tissue specificity on the current X and autosomes (Methods). We find that by far most tissue-specific genes are testis-specific genes, both for the X chromosome and autosomes (Figure 5A). However, the proportion of genes specifically expressed in testis is significantly larger for the X than for autosomes (*p*<0.05, Fisher exact test) (Figure 5A), supporting previous notions [14],[31],[34],[35]. To assess this pattern in more detail, we divided the dataset into genes with 1∶1 orthologs across species (i.e., genes clearly present on the proto-X and autosomes; termed “old” genes in the following) and the remaining genes. The latter set (termed “recent,” for simplification) is thus enriched for new genes or new gene copies that originated through gene duplication or other mechanisms after sex chromosome differentiation (Methods).

Notably, we identify similar proportions of testis-specific genes among the set of old genes for the X and autosomes, whereas there are significantly larger proportions of testis-specific genes for the X than for autosomes among the recent set of X-linked genes (*p*<0.05, Fisher exact test) (Figure 5C). In addition, we find that the extent of testis-specificity on the current X is similar or lower compared to that of the proto-X for the old set of genes (Figure S9). Together, these observations suggest that the excess of testis specificity observed for the X is driven by new genes that accumulated on this chromosome after sex chromosome differentiation, potentially due to the emerging sex-related selective forces [30],[36]. This result is also consistent with previous observations and suggestions [35],[37],[38].

Thus, when removing testis-specific genes from the comparisons of current expression levels on the X and autosomes, X expression levels increase relative to autosomes only for the recent set of genes but not for that of old genes (Figure S10; Table S2). Consequently, the relevant expression level comparisons between (proto) X and autosomes based on 1∶1 orthologous genes presented above are not confounded by the distinct patterns of testis specificity between the two types of chromosomes (Figure S7; Table S2). Notably, recent X-linked genes have overall substantially higher X∶AA ratios than old genes, in particular when testis-specific genes are removed from the analysis (median X∶AA is 0.55 for old genes and 0.80 for recent genes in somatic tissues) (Figure S10; Table S2). Thus, X∶AA ratios that are calculated for all (expressed) X-linked genes (i.e., regardless of the age of genes), as done in most previous studies [11],[14]–[16], reflect more the patterns of recent genes than those of old genes (Figure S10; Table S2), which are the ones relevant for the assessment of dosage compensation. Finally, it is noteworthy that, generally, spatial expression patterns for somatic tissues have been well preserved for the 1∶1 orthologous gene set (Figure S9), which suggests that our X∶pXX ratio estimates are not confounded by major changes in gene functions in somatic tissues since sex chromosome origination.

In summary, our detailed analyses reveal no obvious signal of a global upregulation of X-linked genes after sex chromosome differentiation in eutherians. However, we emphasize that it is well possible that subsets of genes on the X were upregulated, and/or that they were globally upregulated in a subtle manner that does not result in statistically significant signals of upregulation in our analyses.

### Male Versus Female Expression Levels in Marsupials

We then turned to the analysis of dosage compensation patterns in marsupials. Interestingly, median expression levels are very similar between males and females in the organs of the gray short-tailed opossum (*Monodelphis domestica*), a representative of the marsupial lineage (Figure 1). Only in the heart, expression levels are slightly but significantly higher in females (Bonferroni corrected *p*<10^−4^, one-sample Wilcoxon test). Thus, similarly to placental mammals, marsupials evolved efficient dosage compensation mechanisms that led to very similar expression levels between males and females. This observation is surprising in view of previous work that suggested that the *Xist*-independent XCI system in this lineage is incomplete and unstable [21]–[23],[25], but it is consistent with a recent study that revealed efficient XCI for several X-linked genes in *Monodelphis* using fluorescent in situ hybridization analyses [24].

### The Evolution of Dosage Compensation in Marsupials

**Figure 6:**
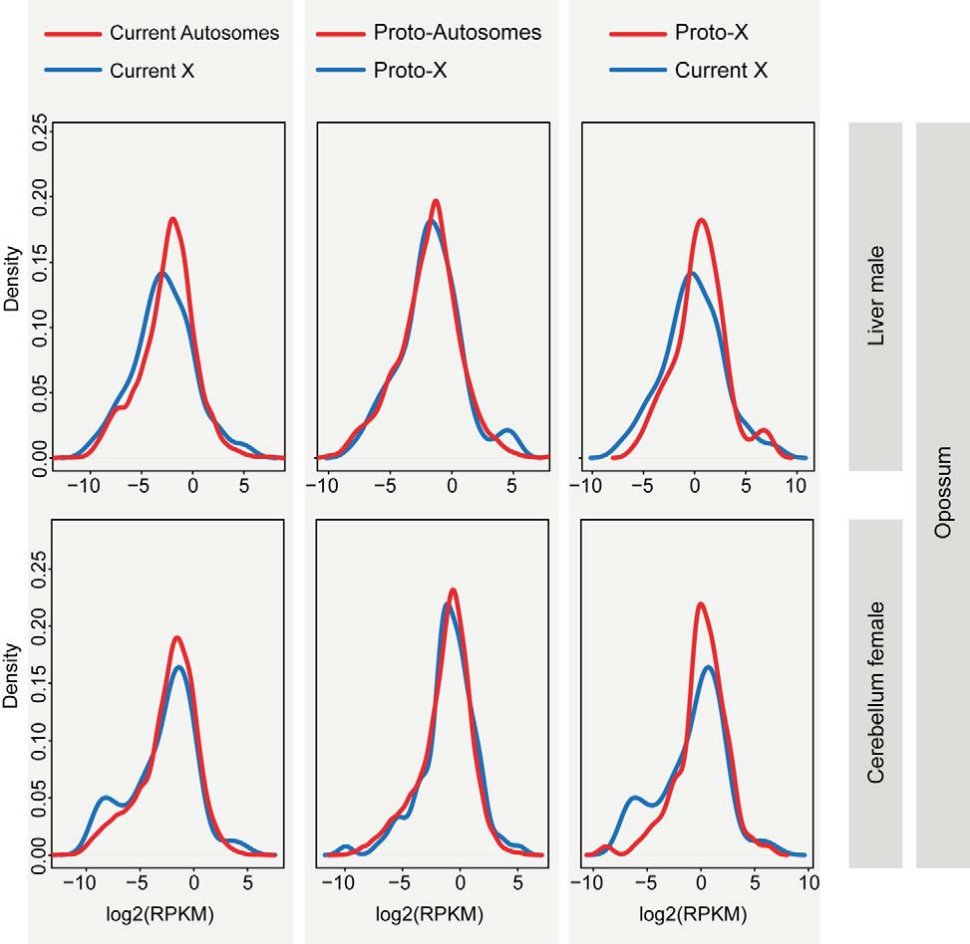
Distributions of current and inferred ancestral expression levels of genes on the marsupial (proto) X chromosomes and autosomes. Distributions of expression levels of (proto) X-linked genes (blue line) and (proto) autosomal genes (red line) are shown for cerebellum and liver from opossum. Expression levels in the comparison of the current X and proto-X (right plots) are normalized by the respective autosomal expression levels. In all cases, the two plotted distributions are not significantly different from each other (Benjamini-Hochberg corrected *p*>0.05; Komolgorov-Smirnov test). See Table S1 for all tests of differences between X and autosomal expression distributions (all tissues).

Remarkably, contrary to the situation in eutherians, we find that the overall expression level distributions of the current marsupial X and therian proto-X are similar in all somatic tissues (Benjamini-Hochberg corrected *p*>0.05, Komolgorov-Smirnov test) (Figure 6; Table S1). Comparisons of X∶pXX ratios confirm that the current opossum X has a relatively similar transcriptional output as the therian proto-X chromosomes in the somatic tissues (median X∶pXX = 0.79) (Figure 3). In some tissues (liver and kidney in both males and females), X∶pXX ratios are not significantly different from 1 (Benjamini-Hochberg corrected *p*>0.05, one-sample Wilcoxon test) but significantly higher than 0.5 (corrected *p*<0.05, one-sample Wilcoxon test). These results are robust to changes in the definition of expressed genes and the removal of testis-specific genes (Figure S7; Table S2). As for eutherians, the latter are only enriched among the recent set of X-linked genes (Figure 5C). Generally, spatial expression patterns have been well preserved for marsupials as well (Figure S9).

**Figure 7:**
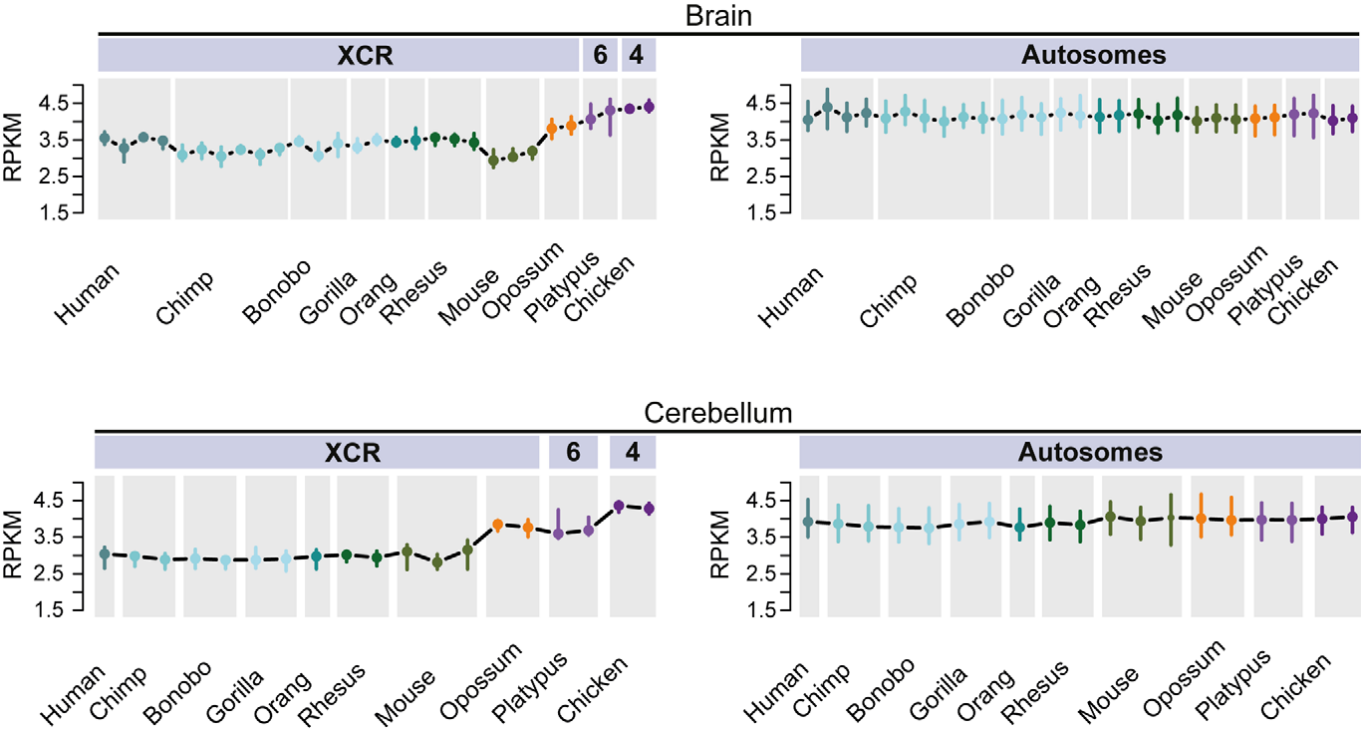
Expression levels of therian genes in the X-conserved region and their autosomal counterparts in platypus and chicken. Left: Global expression levels (based on third quartiles of the RPKM distribution) of genes in the therian XCR (see Figure 3B legend for details) and their autosomal orthologs in outgroup species with different sex chromosome systems (see Figure S10 for all five somatic tissues). Error bars represent the range containing 90% of the third quartiles of individual resampling sets (80% of 90 orthologous genes were resampled 100 times). Right: Expression levels of resampled sets of 90 genes that are autosomal in all ten species. The central value is the median of the third quartiles of resampled sets (error bars represent the central 90% of the distribution of those third quartiles).

Overall, our analyses thus suggest that in contrast to placental mammals, which have the same sex chromosome system (Figure 3A), marsupials appear to have evolved mechanisms that led to partial or full global upregulation of X-linked genes in both sexes. The global difference in XCR expression levels between eutherians and marsupials relative to their autosomal counterparts in platypus and chicken is confirmed and further illustrated by direct comparisons of XCR expression levels that are based on an alternative normalization of our data (i.e., expression values of all orthologous genes were jointly normalized across all species and tissues using a scaling procedure) (Figures 7 and S11) [30]. Finally, we note that the reduced expression of the current opossum X in testis (X∶pXX = 0.39) likely illustrates the consequences of meiotic sex chromosome inactivation (MSCI), which was recently demonstrated to act in marsupials as well [24],[39].

### Male Versus Female Expression Levels in Monotremes

Next, we explored patterns of male versus female expression levels in the egg-laying monotremes, the most basal mammalian lineage. The platypus has five distinct X chromosomes (X_1_–X_5_) [3],[4]. Given that the assembled X_1_ chromosome is largely pseudoautosomal (i.e., it pairs with the Y_1_ chromosome) and that few or no genes have been assigned to chromosomes X_2_ to X_4_ [3],[4],[40],[41], we initially focused our analyses on the X_5_ chromosome, which contains 153 genes in the assembly we utilized and is largely homologous to the Z chromosome of birds (Figure 1) [3],[4].

Our analyses show that X_5_-linked genes are expressed at significantly higher levels in females than in males in all five tissues (median M∶F ratio = 0.59, Bonferroni corrected *p*<0.05, one-sample Wilcoxon test). Notably, we also generated platypus RNA-seq data for fibroblasts (Methods), a cell line previously used to study platypus dosage compensation of individual genes [26]. The fibroblast M∶F ratio (0.68) was not significantly different from those of the other tissues (*p*>0.10, Mann-Whitney *U* test). Consistent with the pseudoautosomal nature of most of the assembled X_1_ chromosome, the majority of X_1_ genes have M∶F ratios close to 1 (Figure 2; Table S3). However, as expected from our observations for the X_5_ chromosome, M∶F ratios for the non-pseudoautosomal region on the long arm of X_1_ (median M∶F ratio = 0.61) are similar to those for the X_5_ chromosome (Figure 2; Table S3). Thus, contrary to therian mammals, monotremes apparently did not evolve efficient mechanisms that equalize expression levels between males and females, which is consistent with the recently reported absence of epigenetic inactivation marks on the platypus X chromosomes [20].

Finally, to explore whether the female-biased expression of non-pseudoautosomal genes on the X_1_ and X_5_ chromosomes reflects that genes on these chromosomes generally have functions that are more important for females, we generated and analyzed RNA-seq data for platypus ovaries. Our analysis of these data show that the extent of ovary-specific expression is very similar and overall low for the two platypus X chromosomes and autosomes (Figure S12). It therefore provides no evidence for an enrichment of female functions on the platypus X chromosomes. For comparison, we performed a similar analysis for the testis, which reveals that only the non-pseudoautosomal part of the X_1_ chromosome is enriched for testis-specific genes (Figure S12). Thus, while genes in this part of the X_1_ are expressed at overall much lower levels in male somatic tissues, it may be slightly enriched for genes with testis functions.

### The Evolution of Dosage Compensation in Monotremes

**Figure 8:**
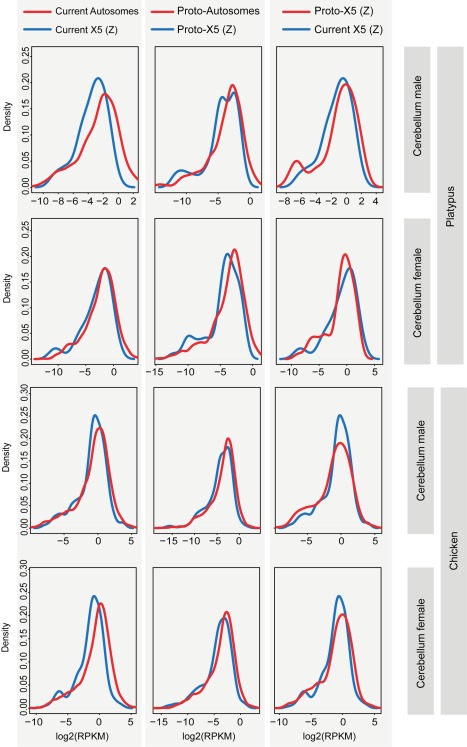
Distributions of current and inferred ancestral expression levels of genes on the platypus (proto) X chromosomes/autosomes and chicken (proto) Z chromosome/autosomes. Distributions of expression levels of (proto) sex chromosome-linked genes (blue line) and (proto) autosomal genes (red line) are shown for cerebellum from platypus and chicken. Expression levels in the comparison of the current X (Z) and proto-X (Z) are normalized by the respective autosomal expression levels (rightmost plots). See Table S1 for all tests of differences between X (Z) and autosomal expression distributions (all tissues).

Expression level distributions of the present-day X_5_ and proto-X_5_ in male platypus are overall very similar (Benjamini-Hochberg corrected *p*>0.05, Komolgorov-Smirnov test) (Figure 8; Table S1) and the median X_5_∶pX_5_X_5_ value across somatic tissues is 0.67. In two tissues (liver and kidney), X_5_∶pX_5_X_5_ ratios are significantly larger than 0.5 (Benjamini-Hochberg corrected *p*<0.05, one-sample Wilcoxon test). Thus, our analyses of X_5_ expression evolution in platypus suggest that this chromosome has become partially upregulated in males (the heterogametic sex) at least in several tissues after sex chromosome differentiation (Figure 3B). Our analyses of females (the homogametic sex) suggest that the X_5_ expression output was essentially preserved during evolution (median X_5_X_5_∶pX_5_X_5_ = 1.07) (Figure 3B). Together, our observations may thus indicate that not only has the X_5_ dosage reduction upon monotreme sex chromosome formation been partially compensated by an upregulation of genes on this chromosome, but that this upregulation is specific to males. Alternatively, the X_5_ upregulation mechanism is not specific to males, but the partial overexpression of genes on these chromosomes in the homogametic sex is avoided by some form of X_5_ inactivation that restores the ancestral X_5_ to autosome balance in this sex. The latter scenario may be less likely, given that chromosome-wide epigenomic marks indicative of global inactivation mechanisms could so far not be detected for the X chromosomes in platypus [20]. We finally note that the preservation of ancestral X_5_ expression levels in females is consistent with the notion (see above) that the higher expression levels of X_5_ genes in this sex relative to males are not reflecting the evolution of new female functions after sex chromosome differentiation but are only due to the X_5_ dosage reduction in males.

### The Evolution of Dosage Compensation in Birds

Our RNA-seq data analyses from non-domesticated chicken (*Gallus gallus*, red jungle fowl) reveal significantly higher expression levels in males (ZZ) than females (ZW) in the five tissues (Figure 1), consistent with the view that birds lack global dosage compensation mechanisms that would lead to similar expression levels between the sexes [27],[28],[42].

Interestingly, our analyses of expression evolution in chicken suggest that the chromosomes ancestral to the Z chromosome already had relatively low expression levels (median pZZ∶pAA = 0.74), which are significantly lower in nearly all tissue comparisons than those inferred for ancestral autosomes (Figure 3B). This observation is interesting in light of the theory that these ancestral sex chromosomes [3], which thus apparently had particular properties in terms of gene expression, represent the ancestral sex chromosome system of amniotes. Notably, similarly to the X_5_ chromosome in platypus, the Z chromosome has become significantly upregulated in all tissues of female chicken (the heterogametic sex) after sex chromosome differentiation (median Z∶pZZ = 0.74; ratios significantly larger than 0.5, one-sample Wilcoxon test) (Figure 3B). However, the expression output of the current Z remains significantly lower than that of the proto-Z chromosomes (Z∶pZZ<1, *p*<0.05, one-sample Wilcoxon test) (Figure 3B), leading to significant differences in overall expression level distributions for four out of five tissues (i.e., brain, cerebellum, heart, liver; corrected *p*<0.05, Komolgorov-Smirnov test) (Figure 8; Table S1). By contrast, the Z expression output in male chicken (the homogametic sex) was essentially preserved during evolution (median ZZ∶pZZ = 1.03 for chicken male) (Figure 8; Table S1). Thus, similarly to the situation in monotremes, these observations might indicate that the Z dosage reduction has been compensated by partial upregulation of genes on this chromosome specifically in the heterogametic sex (i.e., female chicken). Alternatively, they might indicate that the Z upregulation mechanisms are not specific to females, but that the overexpression of genes on these chromosomes in the males is avoided by some form of Z inactivation that restores the ancestral Z to autosome balance in this sex. However, similarly to platypus, chromosome-wide epigenomic marks indicative of global inactivation mechanisms could so far not be detected for these chromosomes in chicken [27], which may render the latter scenario less likely.

Notably, a refined analysis revealed that a subset of genes with Z∶pZZ expression levels close to 1 in female chicken (i.e., genes that apparently have been two-fold upregulated relative to ancestral levels) show median ZZ∶pZZ expression ratios of 1.13 to 1.56 in male somatic organs, which suggests that the upregulation of these genes is not completely specific to the heterogametic sex but affected the homogametic sex to some extent (Figure S13). A similar pattern is observed in platypus, although these results are less clear due to the relatively low number of X_5_-linked genes that could be analyzed (Figure S13). The fact that upregulation was not complete in the homogametic sex for this subset of genes suggests that either the transcriptional upregulation mechanism is more efficient in the heterogametic sex (i.e., it is largely sex specific), or that some form of secondary regulatory buffering/inactivation mechanisms (e.g., regulatory feedback loops, local epigenetic modifications) partially reduce expression levels of these X_5_/Z-linked genes in the homogametic sex of these species.

Overall, our observations that the dosage compensation mechanism in birds and platypus only mildly affected the homogametic sex in these species provide a compelling potential explanation for why evolution of X_5_ and Z inactivation mechanisms was not required in these lineages. The partial and largely sex-specific compensation for the X_5_ and Z dosage reduction in the heterogametic sex also provides an explanation for the only partially sex-biased expression in platypus and birds (i.e., M∶F expression ratios>0.5 and <2, respectively; see above) (Figure 1).

### Dosage Compensation through Downregulation of Functionally Cooperating Autosomal Genes

The analyses described above suggest that, at least in some amniote lineages, dosage reductions resulting from sex chromosome differentiation processes have been compensated by transcriptional upregulations of, at least, subsets of genes. One could argue that X and Z chromosomal genes that have not become upregulated simply represent haplosufficient genes that are insensitive to dosage alterations (e.g., because they lack functional interactions with autosomal genes). Haploinsufficient genes were indeed recently inferred to be underrepresented on the therian X chromosome [43], which may explain why dosage compensation has not been necessary for a number of X-linked genes. However, collectively, dosage insensitivity is unlikely to explain the pattern observed for therian mammals, given that marsupial X-linked genes show strong signatures of upregulation, whereas their eutherian orthologs—derived from the same ancestral genes—do not. Thus, we hypothesize that expression level reductions of dosage sensitive (haploinsufficient) sex chromosomal genes may also have been compensated in other ways.

Given that a main driving force behind the evolution of dosage compensation is likely the maintenance of the balance between X-linked and autosomal gene expression [9], we hypothesized that instead of upregulation of X-linked genes, autosomal genes that functionally interact with X-linked genes could have been downregulated. This mechanism might be more likely for many genes, given that transcriptional upregulation of ancestral genes with already high expression levels may be mechanistically constrained [44], while regulatory mutations leading to transcriptional downregulation may have been fixed more easily during evolution.

**Figure 9:**
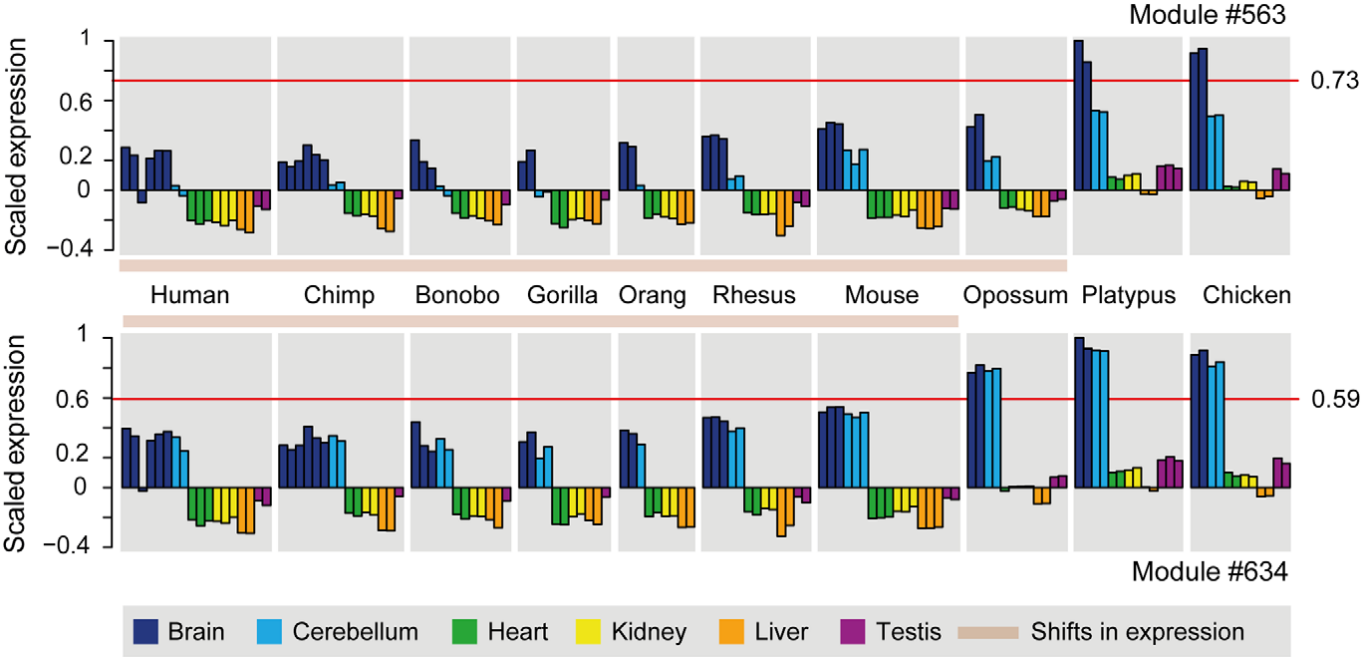
Concerted downregulation of X-linked and autosomal genes in the brain of placental and marsupial (i.e., therian) mammals. Modules with specific expression states in the therian brain (module 563; 330 genes) or eutherian brain/cerebellum (module 634; 313 genes) are shown. Bars represent the weighted average expression of all genes in a module, for each sample (horizontal grey line, average bar height). The horizontal red line represents the cutoff of the biclustering algorithm; samples above the red line are considered to have a distinct expression state. Note that the modules shown are highly enriched for X-linked genes (module 563: 25 observed versus 8.5 expected, *p*<10^−3^; module 634: 28 observed versus 8.3 expected, *p*<10^−4^), as are modules 421, 507, 521, and 618, which display transcriptional downregulations in therians or eutherians and were all considered in the protein–protein interaction analyses (see main text and Methods). All modules can be explored in a searchable database: http://www.unil.ch/cbg/ISA/species.

Remarkably, in the framework of analyses that are fully presented in a previous study [30], we identified drastic and concerted expression level reductions of X-linked and autosomal genes in several organs (brain and cerebellum) that apparently occurred shortly after the differentiation of sex chromosomes in the common therian and eutherian ancestors (Methods) (Figure 9). To explore whether these concerted expression shifts indeed reflect downregulations of autosomal genes in response to dosage reductions of functionally cooperating X-linked partners, we performed protein–protein interaction network analyses in human and mouse, given that protein interaction represents one major way in which genes can functionally cooperate and because this type of cooperation can be assessed with available data for these species.

To do these analyses, we first generated two sets of interacting genes (Methods). Set 1 contained all X-linked genes whose expression levels dropped in the common therian/eutherian ancestor and all autosomal genes that functionally interact with them at the protein level. Set 2 contained all X-linked genes whose expression levels did not drop in the common therian/eutherian ancestor and all autosomal genes that functionally interact with them at the protein level. We then compared the proportion of autosomal partners that have become downregulated in the therian/eutherian ancestor in these two sets. Our analyses revealed a significantly higher proportion of downregulated autosomal genes in set 1 compared to set 2 (*p*<0.02 and *p*<0.05 in the human and mouse analyses, respectively, Fisher exact test) (Table 1). These results suggest that the drop in expression of X-linked genes after sex chromosome differentiation was accompanied by a reduction of expression of a subset of interacting autosomal partner genes.

**Table 1:** Protein interaction networks of X-linked and autosomal genes and evolutionary expression change.

Genes Considered	Human		Mouse	
	Downregulated Interacting Autosomal Genes	Non-downregulated Interacting Autosomal Genes	Downregulated Interacting Autosomal genes	Non-downregulated Interacting Autosomal Genes
**X-linked genes with decreased expression in therians/eutherians**	10 (12.7%)	69 (87.3%)	8 (13.1%)	53 (86.9%)
**Other X-linked genes**	19 (4.9%)	372 (95.1%)	16 (5.1%)	299 (94.9%)
**Autosomal background** a	226 (6.2%)	3,423 (93.8%)	210 (6.2%)	3,183 (93.8%)

a Proportion of downregulated and non-downregulated genes among all autosomal genes in our amniote 1∶1 orthologous gene set for which protein interaction data were available.

This analysis therefore supports our hypothesis that the dosage balance of X-linked and autosomal genes after the attrition of the Y has at least partially been restored by downregulations of autosomal genes that functionally cooperate with genes on the X in one way or another (e.g., through protein–protein interactions but also through other mechanisms, such as functions in the same metabolic pathway, which remain to be assessed). Importantly, the fact that autosomal downregulations have also affected females (Figure 9) may explain why XCI evolved in eutherians in spite of the apparent overall lack of upregulation of X-linked genes after Y decay. In this scenario, the reduced expression of autosomal genes drove the evolution of XCI to restore the X-autosomal dosage balance in eutherian females.

### Other Potential Compensation Mechanisms

In addition to upregulations of individual dosage sensitive genes, dosage insensitivity of certain genes, and/or autosomal downregulations of subsets of X/Z-linked genes, there are at least three other possible explanations for why (complete) global X or Z upregulation was not required after sex chromosome differentiation in amniotes. First, sex-related selective forces may have reshaped functions of certain X-linked genes after sex chromosome differentiation, which could have rendered maintenance of ancestral dosage balances between the X and autosomes unnecessary for these genes. Our analyses described above suggest that spatial expression patterns and hence gene functions may have remained overall conserved on amniote sex chromosomes (Figure S9). However, gene expression evolution was accelerated during the early evolution of the therian X chromosome [30], which potentially reflects functional changes of a subset of genes, but could also indicate upregulations of individual genes in males upon Y decay. Second, we hypothesized that duplication of X-linked genes might constitute a rather straightforward means to reconstitute their ancestral gene dosage in males after the attrition of the Y. Consistent with previous work [38],[45], we indeed detect an accelerated gain of X-linked gene duplicates after therian sex chromosome birth (Figure S14). However, given that these X-linked duplicates tend to be expressed in sex-specific tissues [38],[45] and show substantial divergence at the protein sequence level (Figure S14), these duplicates are unlikely to be linked to the evolution of dosage compensation but probably arose in the framework of a burst of functional adaptation during early X evolution (see above) [38],[45]. Finally, we note that it is possible that some X-linked genes regained their original dosage by relocating to autosomes, a scenario that remains to be investigated.

## Discussion

Our evolutionary analyses of tissue transcriptomes from species representing all major mammalian lineages and birds provide an unprecedented overview of the evolution of dosage compensation in amniotes and fundamental novel insights into the underlying driving forces (Table 2). Our comparisons of current and inferred ancestral expression levels, suggest that the dosage reduction of the therian X upon the attrition of the Y has not been compensated by a measurable global upregulation of genes on the eutherian X. However, our results do not rule out a partial or full upregulation of individual X-linked genes, which was recently suggested to occur for genes encoding components of large protein complexes on the basis of current human X to autosome expression level comparisons (without consideration of the age of the analyzed genes) [32]. Our findings also do not exclude a subtle global upregulation of the X. In any event, they do not provide evidence for a global two-fold upregulation of the X, as originally proposed by Ohno [1]. Thus, our evolutionary analyses resolve recent contradictory studies that were based on analyses of expression levels of genes on the present-day X and autosomes [13],[14],[16],[17],[32]. Furthermore, our analyses suggest that the dosage balance between the X and autosomes, at least for a subset of genes, was instead restored by downregulation of functionally associated autosomal genes after sex chromosome differentiation. X-linked genes that were not compensated by individual upregulations or downregulations of autosomal partners were probably either haplosufficient, obtained new (potentially sex-specific) functions, or were relocated to autosomes. Notably, XCI in eutherian females may have in part evolved as a response to the autosomal downregulation, which also affected females. Thus, our findings revise long-held hypotheses regarding the driving forces underlying eutherian XCI and the associated equilibration of expression levels between males and females. It will be interesting to follow up on various aspects of our eutherian findings in the future. For example, it will be interesting to assess whether the previously reported enrichment of chromatin markers associated with transcriptional activity on the X relative to autosomes [14],[16] is relevant for genes that were already present on the proto-X and would thus explain any potential upregulation of such genes compared to ancestral states.

**Table 2:** Overview of the observed patterns, mechanisms, and evolution of dosage compensation in mammals and birds.

Lineage	Sex Chromosome System^a^	Original Dosage Compensation Mechanism		Global X (Z) Inactivation In Homogametic Sex	Male/Female Expression on X or Z
		X (Z) Upregulation	Detected Alternative Mechanisms		
**Placentals**	XY	None	Downregulation of interacting autosomal genes	Yes	No global sex-bias
**Marsupials**	XY	Partial to complete	Not assessed	Yes	No global sex-bias
**Monotremes**	(XY)_5_	None or partial (largely male-specific)	Not assessed	No	Strongly female-biased
**Birds**	ZW	Partial (largely female-specific)	Not assessed	No	Partially male-biased

a Placental mammals and marsupials have homologous sex chromosomes; monotremes and birds have partially homologous sex chromosomes (see Figures 1 and 3 for details).

Surprisingly, in marsupials, whose sex chromosomes are homologous to those of eutherians, significant upregulation of X-linked genes has occurred. Thus, contrary to eutherians, the evolution of female XCI in marsupials was probably largely driven by global X upregulation, which affected both sexes. Notably, contrary to previous inferences [21]–[23] but consistent with a recent study [24], we find X expression levels to be as similar between marsupial males and females as between the two eutherian sexes. This result suggests that the paternally imprinted XCI mechanism in marsupials, which is thought to represent the ancestral system also in eutherians, is very efficient, probably as efficient as the random XCI that evolved in eutherians. Generally, we note that the fact that eutherians and marsupials show very different and apparently independent responses to the X dosage reduction supports the notion that therian sex chromosomes emerged just prior to the split of these two lineages [2].

In the two other amniote lineages studied here, monotremes and birds, which also have homologous sex chromosomes, the dosage reduction of the X and Z chromosome that arose after sex chromosome differentiation in these lineages, respectively, was compensated in yet another way: the sex chromosomes seem to have become rather specifically upregulated in the heterogametic sex in these lineages. This largely sex-specific upregulation provides a plausible explanation for why the evolution of global inactivation mechanisms of these chromosomes in the homogametic sex was not required in these lineages.

Altogether, our data also shed new light on the long-standing hypothesis that male-heterogametic (XY) systems are more prone to evolve efficient dosage compensation mechanisms than female-heterogametic (ZW) systems [9],[46],[47]. Although the precise extent of the different dosage compensation mechanisms still remains to be established, our data overall suggest that the original sex chromosome dosage reductions have been compensated at least as much in the female-heterogametic birds as in the male-heterogametic mammals. However, as the bird dosage compensation mechanism is nevertheless only partial and has been largely restricted to the heterogametic sex (females), female birds have overall lower transcriptional output from the Z chromosome than males, which was previously interpreted as an overall lack of global dosage compensation in birds, in particular when compared to mammals [27],[28],[42]. Thus, in spite of a strong original dosage reduction response, birds have evolved overall different dosage balances between the Z and autosomes in the two sexes.

In the context of contrasting XY and ZW systems, it is also important to note that the monotreme platypus, whose X_5_ chromosome is largely homologous to the bird Z chromosome, shows similar dosage compensation patterns as birds. Overall, our work therefore suggests that patterns of sex chromosome dosage compensation might mainly depend on the properties of different proto-sex chromosomes and/or potentially fortuitous events and mechanisms that arose soon after sex chromosome differentiation (e.g., sex chromosome up- or autosomal downregulations, specific to the heterogametic sex or not), which determine the evolution of subsequent mechanisms such as sex chromosome inactivation in the homogametic sex. Our results are thus in agreement with a recent hypothesis [48]. Our findings highlight that the evolutionary pressures imposed by sex chromosome dosage reductions in amniotes were resolved in strikingly different ways in the different lineages, even for the same ancestral sex chromosomes.

## Methods

### RNA-Seq Data

Most RNA-seq data were derived from a parallel study, and data production details are provided in this paper [30]. In addition, we generated strand-specific RNA-seq data for male and female platypus fibroblasts as well as RNA-seq data for platypus ovary, essentially on the basis of the Directional mRNA-Seq Library Prep Pre-Release Protocol from Illumina. These sequencing data are available at NCBI Gene Expression Omnibus (GEO) (http://www.ncbi.nlm.nih.gov/geo/) (accession number: GSE36120). Detailed information on genome annotation refinements (all based on Ensembl release 57) [49], as well as RNA-seq read processing and mapping are provided in Brawand et al. [30].

### Sets of Genes

In addition to complete sets of Ensembl protein-coding genes (release 57) for the different species, we specifically identified 1∶1 orthologous genes for each pair of species in our set from the Ensembl database, release 57 [49]). From these pairwise orthology relationships, we then extracted 5,997 gene families that have 1∶1 orthology relationships between all the ten species in our set. This 1∶1 ortholog gene set was used for all cross-species gene expression comparisons (see also below). Specific numbers of (expressed) X (Z) chromosomal genes in the various analyses are provided in the respective figure legends.

### Expression Levels and Normalization

Gene expression values were retrieved from our previous study [30]. In that study, standard RPKM (reads per kilobase of exon model per million mapped reads) expression values [50] (that were then log_2_-transformed) were calculated for each gene. Similarly to this previous study, we then normalized these log_2_-transformed expression values across tissues, or across both tissues and species (for the XCR cross-species comparison) (Figure 7), using a median scaling procedure, to render the data comparable among samples [30]. Specifically, among the genes with expression values in the inner quartile range, we identified the 1,000 genes that have the most conserved ranks among samples and assessed their median expression levels in each sample. We then derived scaling factors that adjust these medians to a common value. Finally, these factors were used to scale expression values of all genes in the samples. The validity of this approach was demonstrated previously [30]. In addition to this general normalization, we normalized X (Z) expression levels relative to autosomal background levels, as described in detail for each specific analysis below.

### Comparisons of Male and Female Expression Levels

Male to female (M∶F) expression level ratios of X(Z)-linked genes were normalized by the median autosomal M∶F expression ratios, given that no global sex-bias is expected for autosomal genes. The absence of a global sex bias among autosomal genes was confirmed by the fact that repeatedly resampled subsets of autosomal genes (corresponding to 80% of the number of X(Z)-linked genes in the respective species) displayed median M∶F ratios close to 1 (log_2_ = 0) (Figure S15A). The variability of M∶F ratio estimates was assessed by resampling corresponding numbers of X (Z) and autosomal genes and then computing the range of the 95% confidence intervals for all tissues in all species (Figure S15A and S15B). The higher variability of M∶F ratios for the eutherian X is at least in part explained by the lower expression levels of X-linked genes in this lineage, which leads to a higher technical variance (i.e., smaller number of mapped RNA-seq reads; see also Methods “Assessment of Technical Noise”) (Figure S15B). Statistically significant deviations of median M∶F ratios from key reference values (i.e., M∶F ratio = 1 [log_2_ = 0], the expectation for no sex-biased expression; M∶F = 0.5 [log_2_ = −1], two-fold higher expression in males; M∶F = 2 [log_2_ = 1]), two-fold higher expression in females] were assessed using one-sample Wilcoxon signed rank tests. *p*-Values were Bonferroni corrected for the total number of tests performed across species and tissues per reference value.

### Assessment of Current Chromosomal Expression Levels

First, we assessed current X(Z) to autosome expression ratios for all expressed genes in a given tissue on the basis of the medians of expression levels of all X(Z)-linked expressed genes and of all autosomal expressed genes (Figure S2). For all further analyses, current X(Z) to autosome expression ratios were based on the medians of expression levels of expressed X(Z)-linked genes with 1∶1 orthologs across all ten species and of expressed autosomal genes whose 1∶1 orthologs are located on autosomes in all ten species (Figures 3B, left, and S3).

### Assessment of Ancestral Chromosomal Expression Levels

The proto-X(Z) to proto-autosomes expression ratios (Figures 3B, middle, and S5) were based on ancestral X(Z) and autosomal expression levels inferred through 1∶1 orthologous genes in outgroup species (i.e., species with non-homologous sex chromosomes) (Figure 3A). Specifically, ancestral expression levels of X(Z)-linked genes (i.e., expression levels of proto-X(Z) chromosomes) for a given species were estimated on the basis of the median expression levels of expressed autosomal 1∶1 orthologs in outgroup species with non-homologous sex chromosomes (Figure 3A). Similarly, ancestral expression levels of autosomal genes in a given species (i.e., expression levels of proto-autosomes) were estimated on the basis of the median expression levels of 1∶1 orthologs that are autosomal in all outgroup species with non-homologous sex chromosomes.

### Assessment of X (Z) to Proto-X (Z) Expression Levels

Current X(Z) to proto-X(Z) expression ratios (Figures 3B, right, and S5) were calculated in the following way. First, we normalized the current expression level value of all expressed X(Z)-linked genes by the median current expression level of a set of 1∶1 orthologous genes that are autosomal in all ten species. We then normalized the ancestral expression level value of each expressed proto-X(Z–linked genes (computed as described above) by the median ancestral expression level of a set of 1∶1 orthologous genes that are autosomal in all ten species. Finally, we then computed the ratio of these two values for each gene and assessed the median of these X(Z) to proto-X(Z) ratios. Statistically significant deviations of these medians from key reference values (e.g., 0.5 [log_2_ ratio of −1]; 1 [log_2_ ratio of 0]; and 2 [log_2_ ratio of 1]) were assessed using one-sample Wilcoxon signed rank tests. *p*-Values were Benjamini-Hochberg corrected for the total number of tests performed across tissues for a given species per reference value.

### Tissue-Specific Expression

To define patterns of tissue specificities in nine species (all species except orang-utan, where no testis sample is available), we proceeded in the following way. First, we calculated consensus expression levels for each gene in a given species' tissue on the basis of the median expression levels of this gene across samples available for this tissue. Notably, for the specific spatial expression analysis of platypus, we generated an additional gene expression set that also included ovary (i.e., seven instead of six tissues were included in this analysis). We then performed two analyses of tissue specificity.

In the first analysis, we defined sets of genes specifically expressed in a given tissue in the following way. A gene was considered to be specifically expressed in a given tissue if its consensus expression level (see above) was at least two-fold higher in that tissue than in the other tissues (see Figure 5A for the pattern in humans).

In the second analysis, we defined indices of tissue specificities for each gene by dividing their consensus expression value in a given tissue by the sum of their consensus expression values in all tissues. This index value can thus range from 0 (no expression in that tissue and expression in at least one other tissue) to 1 (only expressed in that tissue). See Figures 5B and S9 for results obtained using this index.

For the specific testis analyses (Figures 5C, S7, and S10), genes were defined as being specifically expressed in testis if the testis specificity index was >0.75. This threshold was based on the distribution of this index shown in Figure 5B and separates the two distinct populations of genes evident in this plot.

### Transcription Modules and X-Autosome Protein Interaction Analyses

In a previous analysis of the data used here [30], we identified groups of genes that show concerted shifts of gene expression levels in subsets of samples (so-called transcription modules). We then selected transcription modules that showed significant enrichments for X-linked genes and a decreased expression in eutherians (identifiers 421, 618, and 634) or therians (identifiers 507, 521, and 563). In these modules, we could thus identify 40 X-linked and 413 autosomal genes whose expression levels decreased at the same time in the common ancestor of therians or eutherians (i.e., soon after sex chromosome origination). We then retrieved protein–protein interaction data for human and mouse from the version 8.3 of the STRING database [51] and identified protein interaction partners for all genes in our set of 5,997 1∶1 orthologs for which protein interaction data were available (3,758 in humans and 3,498 in mouse) (Table 1). Together, these data allowed us to extract two sets of protein–protein interaction gene sets. The type 1 set contained all X-linked genes whose expression levels dropped in the common therian/eutherian ancestor and all autosomal genes that functionally interact with them at the protein level (24 X-linked genes and 79 autosomal interactors in humans; 19 X-linked genes and 61 autosomal interactors in mouse). The type 2 set contained all X-linked genes whose expression levels *did not* drop in the common therian/eutherian ancestor and all autosomal genes that functionally interact with them at the protein level (72 X-linked genes and 391 autosomal interactors in humans; 76 X-linked genes and 315 autosomal interactors in mouse). We then assessed the proportions of autosomal interaction partners that became downregulated in the therian/eutherian ancestor in the two types of gene sets, which revealed a significant excess of autosomal downregulation in the type 1 gene set (see Table 1 and main text for details).

### Patterns of Intrachromosomal Duplications after Sex Chromosome Origination

Mammalian gene duplication data were retrieved from the Ensembl database (release 57). Using a modification of a previous bioinformatics pipeline [2], we identified intronless retroposed gene copies. We removed these retrocopies from the Ensembl gene duplication data, because we considered them unlikely to have contributed to X dosage compensation (e.g., many retrogenes are not functional, do not preserve ancestral expression patterns, and/or do not originate from the chromosome on which their ancestral precursor genes are located). Using Ensembl phylogenetic dating information [49], we then identified, for each branch leading to humans, all distinct paralogy groups with at least one duplication event on that branch. Next, we extracted those paralogy groups for which most of the branch-specific duplication events were intrachromosomal (i.e., >50% of the genes currently being located on the same human chromosome) and then computed, for each branch, the ratios of the number of predominantly X-linked and autosomal paralogy groups, normalized by the number of genes on the current human X chromosome and autosomes, respectively (Figure S14). The ratios of the median protein sequence identity for gene duplicates (based on pairwise identity values extracted from the Ensembl database) on the X or autosomes in the respective paralogy groups were also calculated for each branch (Figure S14).

### Old Versus Recent Genes

For all evolutionary analyses, we used the set of 5,997 1∶1 orthologous genes described above. These genes represent “old” genes that were already present in the common amniote ancestor and therefore were already present on the proto-sex chromosomes and proto-autosomes. We also performed separate analyses for the remaining genes (termed “recent” in the main text for simplification), which are thus expected to be enriched for genes that emerged more recently in amniotes through gene duplication or other origination mechanisms, although this set potentially also contains ancient paralogous gene copies for which 1∶1 orthologous relationships cannot be unambiguously determined. To specifically assess the amount of genes that originated by gene duplication since the therian sex chromosome origination on the lineage leading to humans, we extracted from the gene duplication data described in the previous Methods section genes that are part of gene families that experienced at least one duplication event since the separation of the monotreme and therian lineages (sex chromosomes are thought to have originated at some point in the common ancestor of therian mammals, i.e., after the monotreme/therian split) [2],[3]. This analysis shows that 40% of genes in the “recent” set of genes on the human X chromosome are part of families that experienced a duplication event at some point since the divergence of therians and monotremes.

### Assessment of Technical Noise

Due to stochastic variation in the RNA-seq procedure, the observed read coverage for a gene may not directly correspond to the read coverage this gene should theoretically have based on its actual expression level in the sample. The extent of the effect of this stochastic variation in read coverage is expected to be negatively correlated with the actual read coverage of a gene (i.e., genes with lower read coverage are more affected by the stochastic variation inherent in the RNA-seq procedure).

To assess the technical (stochastic) variation in our data, we first performed simulation-based analyses. Specifically, we generated a set of 600 hypothetical genes with an expected actual read coverage ranging from 1 to 600 (this range corresponds to the observed range of median number of reads in our biological samples), resulting in a universe of 180,300 reads. We then performed resampling analyses where 180,300 reads were assigned to each of the 600 genes with probabilities proportional to the expected actual read coverage of each gene. For each resampling set, we computed the variation between the simulated value and the theoretical one using the following formula: (|*t*−*s*|/*t*)*100, where *t* and *s* represent the theoretical and simulated numbers of reads, respectively. For each gene, we computed the median variation value from the 1,000 simulated values and plotted this variation as a function of the theoretical actual number of reads (Figure S16, left). Consistent with the expectation, this plot shows that low read coverage leads to a high impact of technical variation, whereas increasing read coverage gradually reduces this impact.

Our different biological samples have median read coverage that ranges from 28 reads to 512 reads for X/Z-linked genes and from 47 to 536 reads for autosomal genes. Our simulated data suggest that the variation expected for these medians ranges from approximately 3%–12% for X(Z)-linked genes (median of this variation: 7%) and from approximately 3%–9% for autosomal genes (median variation: 5.6%) (Figure S16, right). Notably, the specific ranges of the variation for the eutherian data are very similar (X: 7.1%; autosome: 5.8%). Overall, these results suggest that technical variation is overall relatively low in our assessments of median gene expression levels.

In addition to these simulation-based analyses, we also assessed the extent of technical variation by assessing differences in X(Z)∶AA ratios among technical RNA-seq data replicates (Figure S17). This analysis shows that median X(Z)∶AA ratios are very similar and statistically indistinguishable between replicates; thus, consistent with the simulation-based analysis, this analysis further supports the notion that the technical variance in our data and its impact on the various expression level estimates is overall low.

## Supporting Information

Figure S1 **Median male versus female expression levels of mammalian X-linked and avian Z-linked genes in five somatic tissues.** Median male to female gene expression level ratios for expressed genes are shown for five somatic tissues derived from nine mammals and one bird. Note that values are plotted on a log_2_ scale to allow for linear and symmetrical patterns. Specifically, male and female expression values were compared for the therian XCR (see Figure 1 for ratios based on entire X), platypus X5, and chicken Z chromosome. Numbers of eutherian XCR genes considered: 209 (human), 193 (chimp), 205 (gorilla), 207 (orang), 212 (macaque), 212 (mouse). Statistically significant deviations from the reference values (0.5 [log_2_ ratio of −1]; 1 [log_2_ ratio of 0]; and 2 [log_2_ ratio of 1]), as assessed by one-sample Wilcoxon signed rank tests (Benjamini-Hochberg corrected *p*<0.05) are indicated to the right (orange/blue boxes).
(TIF)

Figure S2 **Median X (Z) to autosome expression level ratios and 95% confidence intervals of all expressed genes (RPKM>0) on the current sex chromosomes in five representative amniotes.**
(TIF)

Figure S3 **Distributions of current expression levels of genes on the eutherian X chromosome and autosomes.** Distributions of expression levels of genes on the current X (blue line) and current autosomes (red line) are shown for cerebellum and XX from human, XX, and XX. X and autosomal distributions are significantly different for human XX tissue, mouse XX, and XX (Benjamini-Hochberg *p*<0.05; corrected Komolgorov-Smirnov test). See Table S1 for all tests of differences between X and autosomal expression distributions (all tissues from all species).
(TIF)

Figure S4 **Current versus ancestral expression levels of autosomal genes.** For each species and tissue, we resampled (100 times) the current to ancestral expression ratio (both normalized by the median expression of non-sampled autosomal genes) for as many autosomal genes as sex chromosome-linked genes among the amniote 1∶1 orthologous gene set. The median expression ratio of all resampling sets and the range containing 90% of the medians of individual resampling sets are shown. Note that values are plotted on a log_2_ scale to allow for linear and symmetrical patterns.
(TIF)

Figure S5 **Current and inferred ancestral expression levels of genes on the mammalian (proto) X or avian Z chromosomes.** Left: median X (Z) to autosome expression level ratios of genes on the current sex chromosomes. Middle: median X (Z) to autosome ratios of genes on “proto-sex chromosomes,” as inferred from autosomal one-to-one orthologous genes from species with non-homologous sex chromosomes (see Figure 3B, main text, and Methods for details). Right: median current to ancestral X (Z)-linked gene expression ratios (normalized by expression levels of autosomal genes, respectively). Statistically significant deviations from the reference values (0.5 [log_2_ ratio of −1]; 1 [log_2_ ratio of 0]; and 2 [log_2_ ratio of 1]), as assessed by one-sample Wilcoxon signed rank tests (Benjamini-Hochberg corrected *p*<0.05) are indicated to the right of each plot (orange/blue boxes).
(TIF)

Figure S6 **Current versus ancestral expression for XCR-linked genes in therians.** Median current to ancestral XCR-linked gene expression ratios (normalized by expression levels of autosomal genes, respectively). Note that values are plotted on a log_2_ scale to allow for linear and symmetrical patterns. Numbers of XCR genes (i.e., genes with clear 1∶1 orthologs across the ten species) considered in these analyses are: 90 (human), 88 (chimp and bonobo), 90 (gorilla), 89 (orang), 89 (macaque), 153 (mouse) and 88 (opossum). See Figure S2 for all X(Z)-linked genes and species. Statistically significant deviations from the reference values (0.5 [log_2_ ratio of −1]; 1 [log_2_ ratio of 0]; and 2 [log_2_ ratio of 1]), as assessed by one-sample Wilcoxon signed rank tests (two-tailed *p*<0.05 after Bonferroni correction for 85 tests, corresponding to the total of individual tests performed per reference value) are indicated to the right of each plot (orange/blue boxes).
(TIF)

Figure S7 **X to proto-X expression ratios for different gene sets (human, mouse, opossum).** Left: X∶pXX ratios calculated for genes with ≥1 read (black circles) or ≥3 reads (red squares) on both the current X and proto-X. Right: X∶pXX ratios for all expressed genes (black circle) or all genes except testis specific genes (red squares). Values are plotted on a log_2_ scale (e.g., 0.5 [log_2_ ratio of −1]; 1 [log_2_ ratio of 0]; and 2 [log_2_ ratio of 1]). See Table S2 for values in all species and tissues.
(TIF)

Figure S8 **X to proto-X expression ratios when excluding various proportions of genes with lower expression levels.** (A) Original X∶pXX ratios (black circles) and 95% confidence intervals (black lines) for all expressed genes are shown. Green circles indicate X∶pXX values calculated for datasets where various proportions of the most lowly transcribed genes for both the current X and proto-X are excluded (see Text S1 for details). Sample abbreviations: br, brain; cb, cerebellum; ht, heart; kd, kidney; lv, liver; ts, testis; M, male; F, female. (B) Comparisons of X∶pXX values based on trimmed and untrimmed data. For each eutherian species studied, the number of times the computed X∶pXX ratios fall within or outside the original 95% confidence intervals are shown.
(TIF)

Figure S9 **Tissue-specificities of genes on the X (Z) and proto-X (Z) chromosomes in amniotes.** Distributions of the individual tissue specificity indices for the X (Z) and proto-X (Z) chromosomes are plotted. Statistical differences between current and proto sex chromosomes, as assessed by paired Mann-Whitney *U* tests (MW), are indicated. Also, the correlations between tissue-specificity indices of current and ancestral genes are indicated (correlation coefficient, *r*). An asterisk indicates statistically significant correlations (*p*<0.05).
(TIF)

Figure S10 **Human and mouse X∶AA ratios for recent and old genes (human, mouse, and opossum).** X∶AA ratios calculated for expressed genes (black circles) or expressed genes except testis-specific genes (red squares) for three different sets of X-linked genes: All expressed genes (left), a set of genes enriched for genes that accumulated since sex chromosome differentiation (“Recent Genes,” middle panels), and 1∶1 orthologs present on the current X and proto-X (“Old” genes, right). See Table S2 for values in all species and tissues.
(TIF)

Figure S11 **Expression levels of therian genes in the X-conserved region and their autosomal counterparts in platypus and chicken.** Left: global expression levels (based on third quartiles of the RPKM distribution) of genes in the therian XCR (see Figure 3B for details) and their autosomal orthologs in outgroup species with different sex chromosome systems (see Figure S7 for all five somatic tissues). Error bars represent the range containing 90% of the third quartiles of individual resampling sets (80% of 90 orthologous genes were resampled 100 times). Right: expression levels of resampled sets of 90 genes that are autosomal in all ten species. The central value is the median of the third quartiles of resampled sets (error bars represent the central 90% of the distribution of those third quartiles). Has, human; Ptr, chimp; Ppa, bonobo; Ggo, gorilla; Ppy, orang; Mml, macaque; Mmu, mouse; Mdo, opossum; Oan, platypus; Gga, chicken.
(TIF)

Figure S12 **Patterns of testis and ovary specificities on the platypus X_1_ and X_5_ chromosomes.** Density plots of ovary and testis specificity indices (Methods) for genes on the platypus X_1_ and X_5_ chromosomes. “Recent genes”: all genes except 1∶1 orthologs present on both the current X and proto-X chromosomes; “Old genes”: 1∶1 orthologs present on both the current X and proto-X chromosomes.
(TIF)

Figure S13 **Sex chromosomal genes strongly upregulated in the heterogametic sex of platypus and chicken.** For each tissue, we defined subsets of X(Z)-linked genes for which current and ancestral expression levels (see Methods for details regarding their calculation) were very similar in the heterogametic sex of chicken and platypus (i.e., log_2_ X∶pXX or Z∶pZZ ratio between 0.71 and 1.41 [−0.5 and 0.5 in a log_2_ scale, respectively]; note that using more stringent log_2_ thresholds of 0.81 and 1.23 [−0.3 and 0.3 in a log_2_ scale, respectively] gave similar results)]. We then assessed the distribution of the extent of upregulation for these subsets of genes in the homogametic sex. Values are plotted on a log_2_ scale to allow for linear and symmetrical patterns.
(TIF)

Figure S14 **Rates of gene duplication on the X and autosomes during amniote evolution and duplicate gene preservation.** The rate of intra-chromosomal protein-coding gene duplication is indicated in black; sequence identity of proteins encoded by the corresponding duplicated genes is indicated in green. See Methods for details on identification and dating of duplication events as well as sequence conservation analysis.
(TIF)

Figure S15 **Gene expression variance on sex chromosomes and autosomes.** (A) Resampling analysis of the male to female (M∶F) expression ratio of sex chromosome-linked and autosomal genes in the ten different species. For both sex chromosome-linked genes and autosomal genes, we resampled (100 times) 80% of the total number of sex chromosome-linked genes for each species and tissue (medians of the upper and lower bounds of the 95% confidence intervals of each resampling set are indicated). Note that values are plotted on a log_2_ scale to allow for linear and symmetrical patterns. (B) Left: distribution of the range of confidence intervals indicated above for sex chromosomes and autosomes, respectively. Right: distribution of the median number of mapped RNA-seq reads for the same sets of sex chromosome-linked and autosomal resampled genes. Statistical differences (*p*-values, p) between the respective distributions, as assessed by Mann-Whitney *U* tests (MW), are indicated.
(TIF)

Figure S16 **Expected random variation of median expression levels in function of number of mapped reads.** Left: The plot shows estimated read sampling variations given theoretical numbers of mapped reads (black curve). Actual median number of reads observed for X-linked (blue lines) and autosomal genes (red lines) in our different biological samples are indicated. See Methods for details of the simulations procedure. Right: Range and median of expected percentage of random variation for the actual median number of reads observed for X-linked and autosomal genes in our different biological samples.
(TIF)

Figure S17 **Variation of X∶AA estimates among technical replicate RNA-seq datasets.** Median X (Z) to autosome expression level ratios and 95% confidence intervals of all expressed genes for six sets of replicate RNA-seq data are shown. The respective sets represent separate lanes of different Illumina GA IIx runs for the same RNA-seq library. The ratios in the respective pairwise comparisons are not significantly different from each other (*p*>0.15, Mann-Whitney *U* test). The number of reads for each lane ranges from 1.7–33 million reads. Notably, the respective datasets for each species' tissue were pooled for the main analyses represented in the paper.
(TIF)

Figure S18 **Effect of increasing expression level thresholds for the calculation of X∶AA ratios.** (A) Effect of increasing expression level thresholds on observed X∶AA ratios for human brain. Separate curves are shown for all expressed genes, recent genes (i.e., all genes except 1∶1 orthologs present on both the current chromosomes and ancestral/proto chromosomes), and old genes (i.e., 1∶1 orthologs present on both the current chromosomes and ancestral/proto chromosomes), respectively. (B) Black curve: random simulation of expression level distribution for autosomal genes, which is based on observed median, standard deviation, and number of expressed autosomal genes in human brain (*AA* set). Green curve: random simulation of expression level distribution for X-linked genes based on observed median and standard deviation of expressed autosomal genes in human brain, and the number of expressed X-linked genes in human brain (*X1* set). This distribution thus reflects a set of X-linked genes whose overall distribution is not different from autosomal genes. Orange curve: same distribution as the one represented by the green curve, but with a two-fold reduction of expression levels for each gene (*X2* set). (C) Effect of increasing expression level thresholds on simulated X∶AA ratios based on simulated expression level distributions such as the ones described in (B). Green curve: the curve represents the median *X1*∶*AA* ratio for 1,000 generated random distributions of *X1* and *AA* expression levels. Orange curve: The curve represents the median *X2*∶*AA* ratio for 1,000 generated random distributions of *X2* and *AA* expression levels. The range covering 90% of the computed ratios is shown by the respective shadings. Together, these results show that gradually increasing expression level thresholds will gradually increase X∶AA ratios and eventually lead to X∶AA ratios around 1 (log_2_ ratio of 0), in spite of actual two-fold lower expression levels of X-linked genes.
(TIF)

Table S1 **X(Z) to autosome, proto-X(Z) to proto-autosome, and X(Z) to proto-X(Z) expression level ratios, as well as associated** ***p*** **-values.**
(XLS)

Table S2 **X(Z) to autosome and X(Z) to proto-X(Z) expression level ratios for different gene sets and filtering methods.**
(XLS)

Table S3 **Male to female expression level ratios for platypus X1 chromosome.**
(XLS)

Text S1 **Discussion of expression cutoffs.**
(DOC)

## Abbreviations

RNA-seq: RNA sequencing
RPKM: reads per kilobase of exon model per million mapped reads
XCI: X chromosome inactivation
XCR: X-conserved region
