# Supplementary figures and images for: Mechanisms and Evolutionary Patterns of Mammalian and Avian Dosage Compensation
Source: PLoS Biol. 2012 May 15;10(5):e1001328. doi: 10.1371/journal.pbio.1001328 (PMC3352821; doi:10.1371/journal.pbio.1001328)

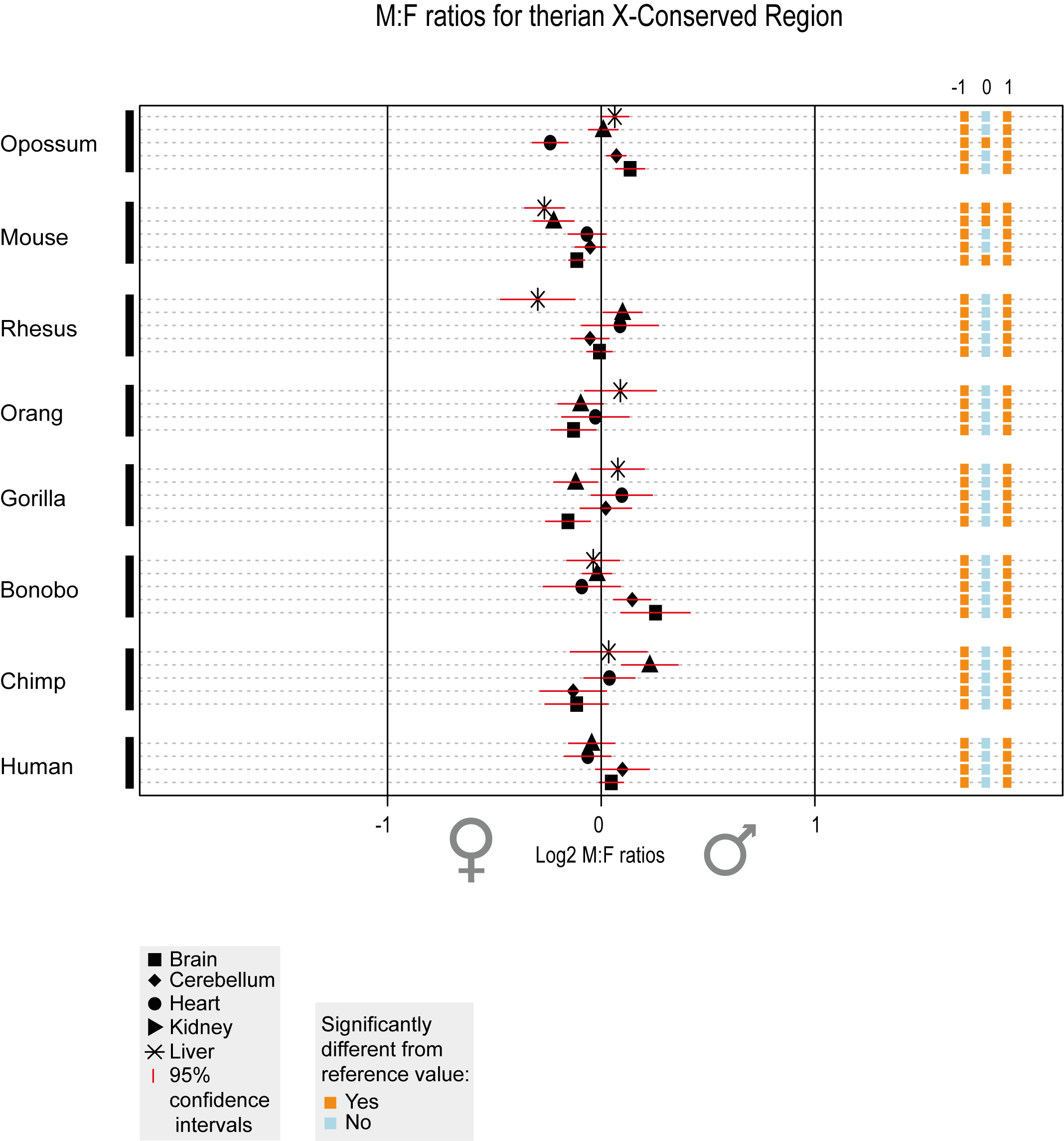

Supplement: Figure S1 — Median male versus female expression levels of mammalian X-linked and avian Z-linked genes in five somatic tissues. Median male to female gene expression level ratios for expressed genes are shown for five somatic tissues derived from nine mammals and one bird. Note that values are plotted on a log2 scale to allow for linear and symmetrical patterns. Specifically, male and female expression values were compared for the therian XCR (see Figure 1 for ratios based on entire X), platypus X5, and chicken Z chromosome. Numbers of eutherian XCR genes considered: 209 (human), 193 (chimp), 205 (gorilla), 207 (orang), 212 (macaque), 212 (mouse). Statistically significant deviations from the reference values (0.5 [log2 ratio of −1]; 1 [log2 ratio of 0]; and 2 [log2 ratio of 1]), as assessed by one-sample Wilcoxon signed rank tests (Benjamini-Hochberg corrected p<0.05) are indicated to the right (orange/blue boxes). (TIF) [file pbio.1001328.s001.tif]

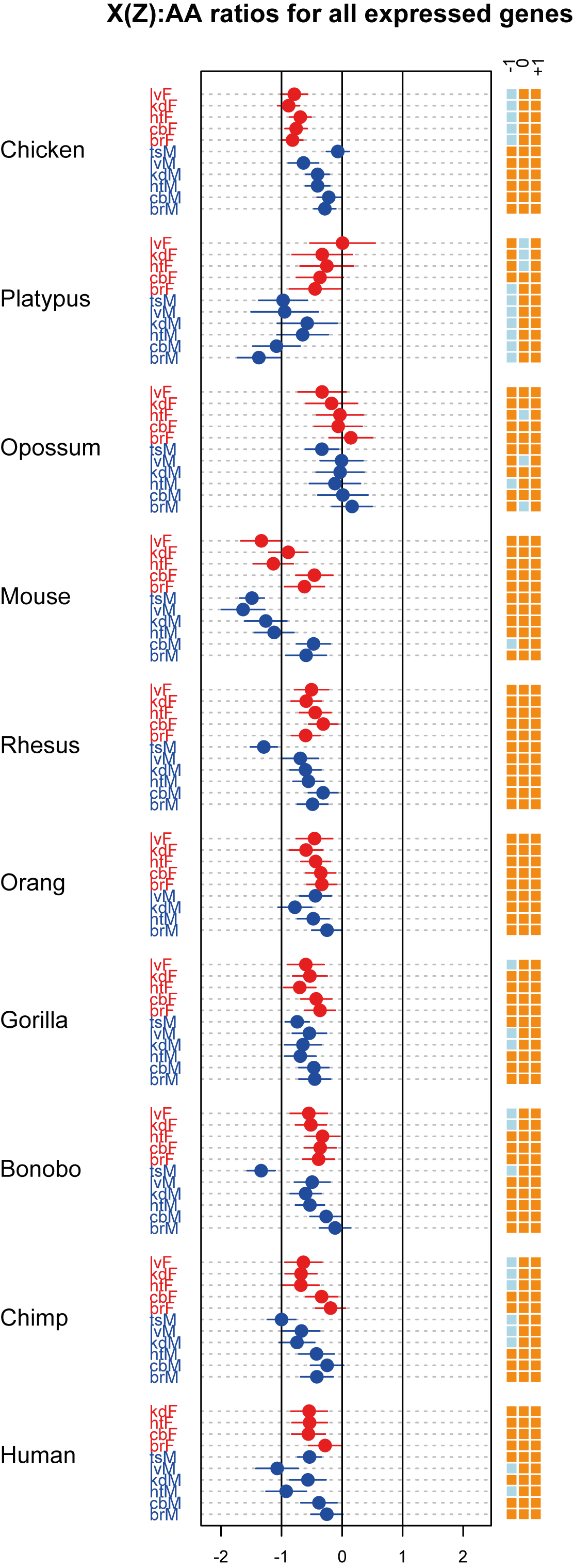

Supplement: Figure S2 — Median X (Z) to autosome expression level ratios and 95% confidence intervals of all expressed genes (RPKM>0) on the current sex chromosomes in five representative amniotes. (TIF) [file pbio.1001328.s002.tif]

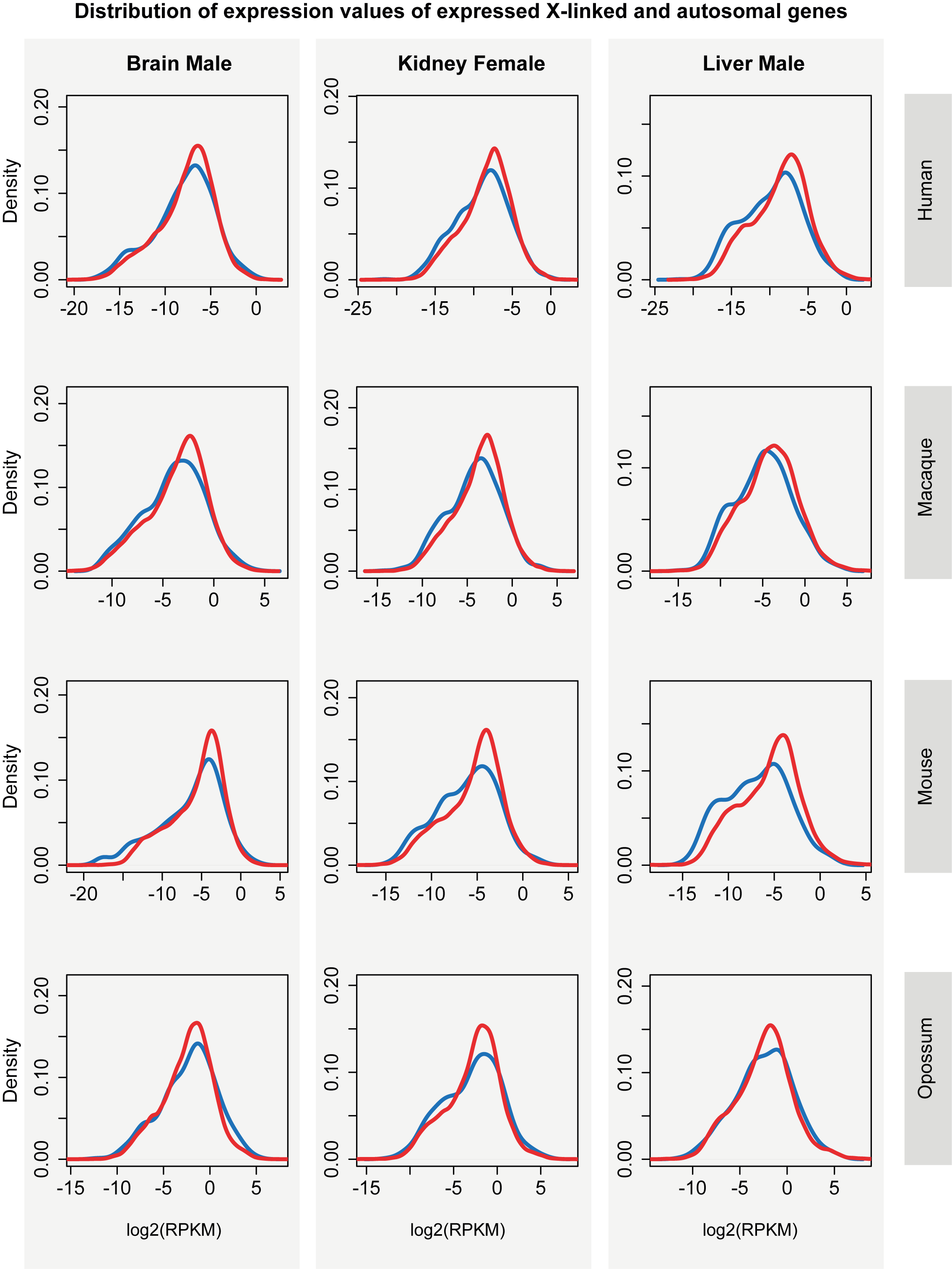

Supplement: Figure S3 — Distributions of current expression levels of genes on the eutherian X chromosome and autosomes. Distributions of expression levels of genes on the current X (blue line) and current autosomes (red line) are shown for cerebellum and XX from human, XX, and XX. X and autosomal distributions are significantly different for human XX tissue, mouse XX, and XX (Benjamini-Hochberg p<0.05; corrected Komolgorov-Smirnov test). See Table S1 for all tests of differences between X and autosomal expression distributions (all tissues from all species). (TIF) [file pbio.1001328.s003.tif]

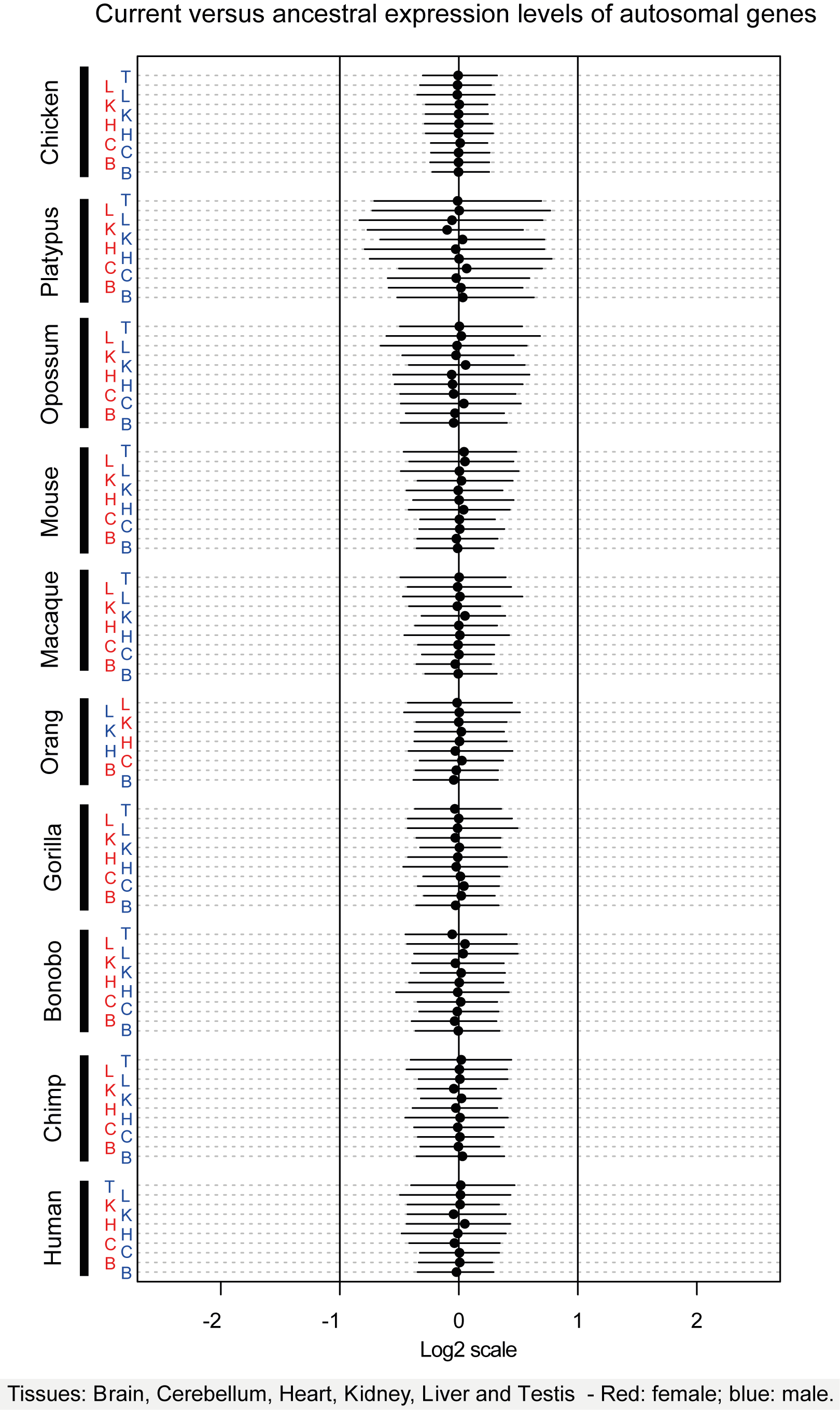

Supplement: Figure S4 — Current versus ancestral expression levels of autosomal genes. For each species and tissue, we resampled (100 times) the current to ancestral expression ratio (both normalized by the median expression of non-sampled autosomal genes) for as many autosomal genes as sex chromosome-linked genes among the amniote 1∶1 orthologous gene set. The median expression ratio of all resampling sets and the range containing 90% of the medians of individual resampling sets are shown. Note that values are plotted on a log2 scale to allow for linear and symmetrical patterns. (TIF) [file pbio.1001328.s004.tif]

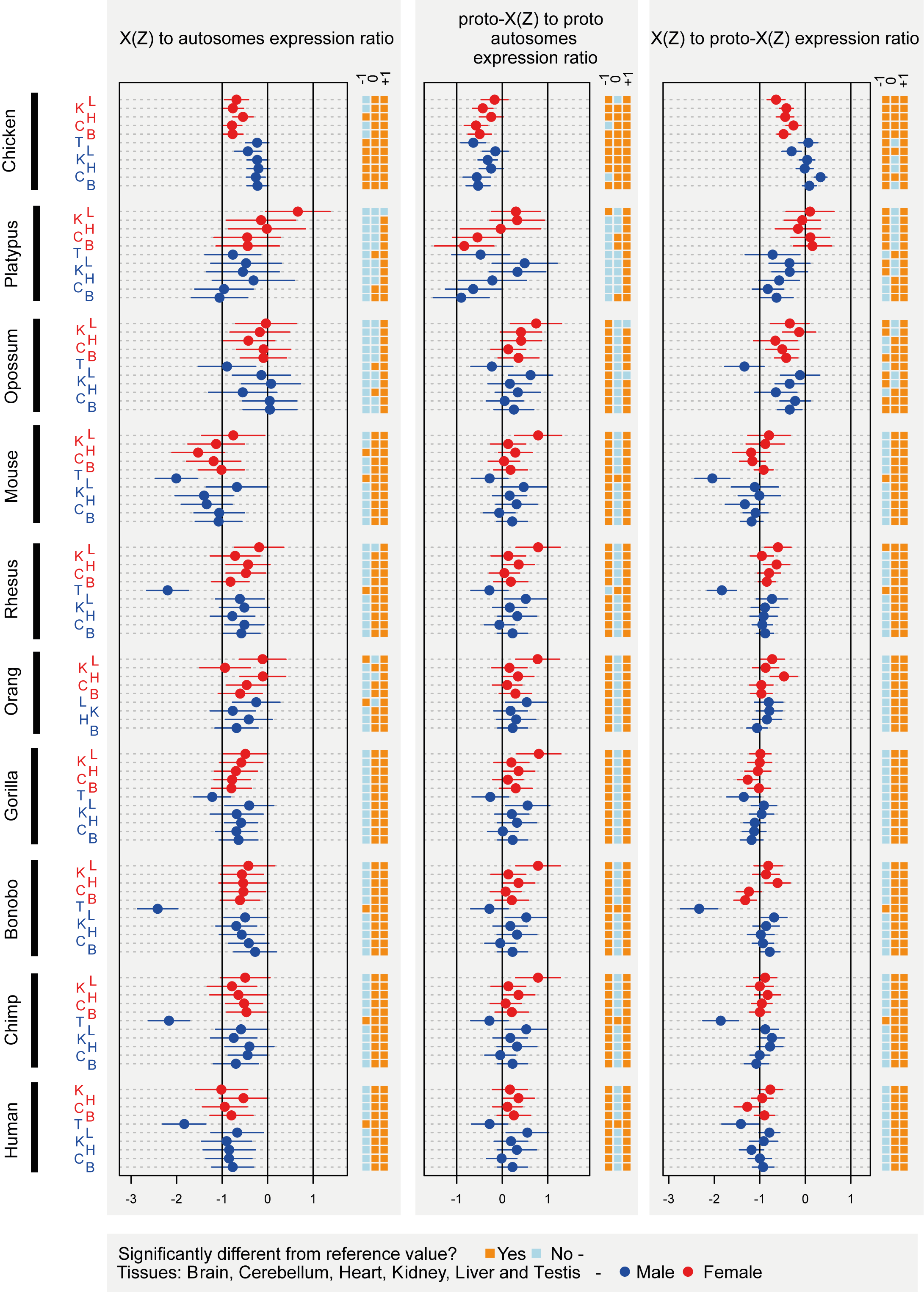

Supplement: Figure S5 — Current and inferred ancestral expression levels of genes on the mammalian (proto) X or avian Z chromosomes. Left: median X (Z) to autosome expression level ratios of genes on the current sex chromosomes. Middle: median X (Z) to autosome ratios of genes on “proto-sex chromosomes,” as inferred from autosomal one-to-one orthologous genes from species with non-homologous sex chromosomes (see Figure 3B, main text, and Methods for details). Right: median current to ancestral X (Z)-linked gene expression ratios (normalized by expression levels of autosomal genes, respectively). Statistically significant deviations from the reference values (0.5 [log2 ratio of −1]; 1 [log2 ratio of 0]; and 2 [log2 ratio of 1]), as assessed by one-sample Wilcoxon signed rank tests (Benjamini-Hochberg corrected p<0.05) are indicated to the right of each plot (orange/blue boxes). (TIF) [file pbio.1001328.s005.tif]

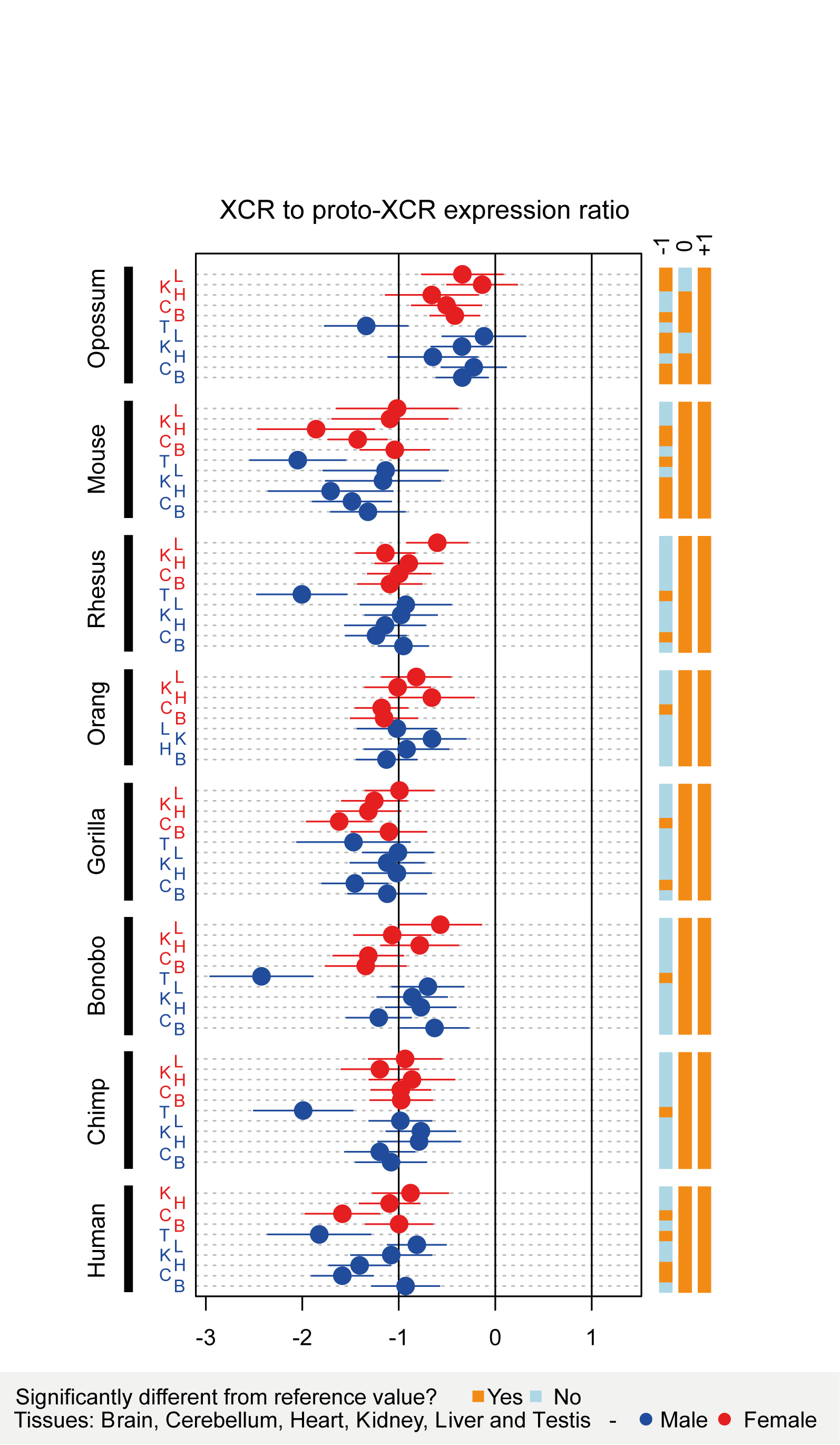

Supplement: Figure S6 — Current versus ancestral expression for XCR-linked genes in therians. Median current to ancestral XCR-linked gene expression ratios (normalized by expression levels of autosomal genes, respectively). Note that values are plotted on a log2 scale to allow for linear and symmetrical patterns. Numbers of XCR genes (i.e., genes with clear 1∶1 orthologs across the ten species) considered in these analyses are: 90 (human), 88 (chimp and bonobo), 90 (gorilla), 89 (orang), 89 (macaque), 153 (mouse) and 88 (opossum). See Figure S2 for all X(Z)-linked genes and species. Statistically significant deviations from the reference values (0.5 [log2 ratio of −1]; 1 [log2 ratio of 0]; and 2 [log2 ratio of 1]), as assessed by one-sample Wilcoxon signed rank tests (two-tailed p<0.05 after Bonferroni correction for 85 tests, corresponding to the total of individual tests performed per reference value) are indicated to the right of each plot (orange/blue boxes). (TIF) [file pbio.1001328.s006.tif]

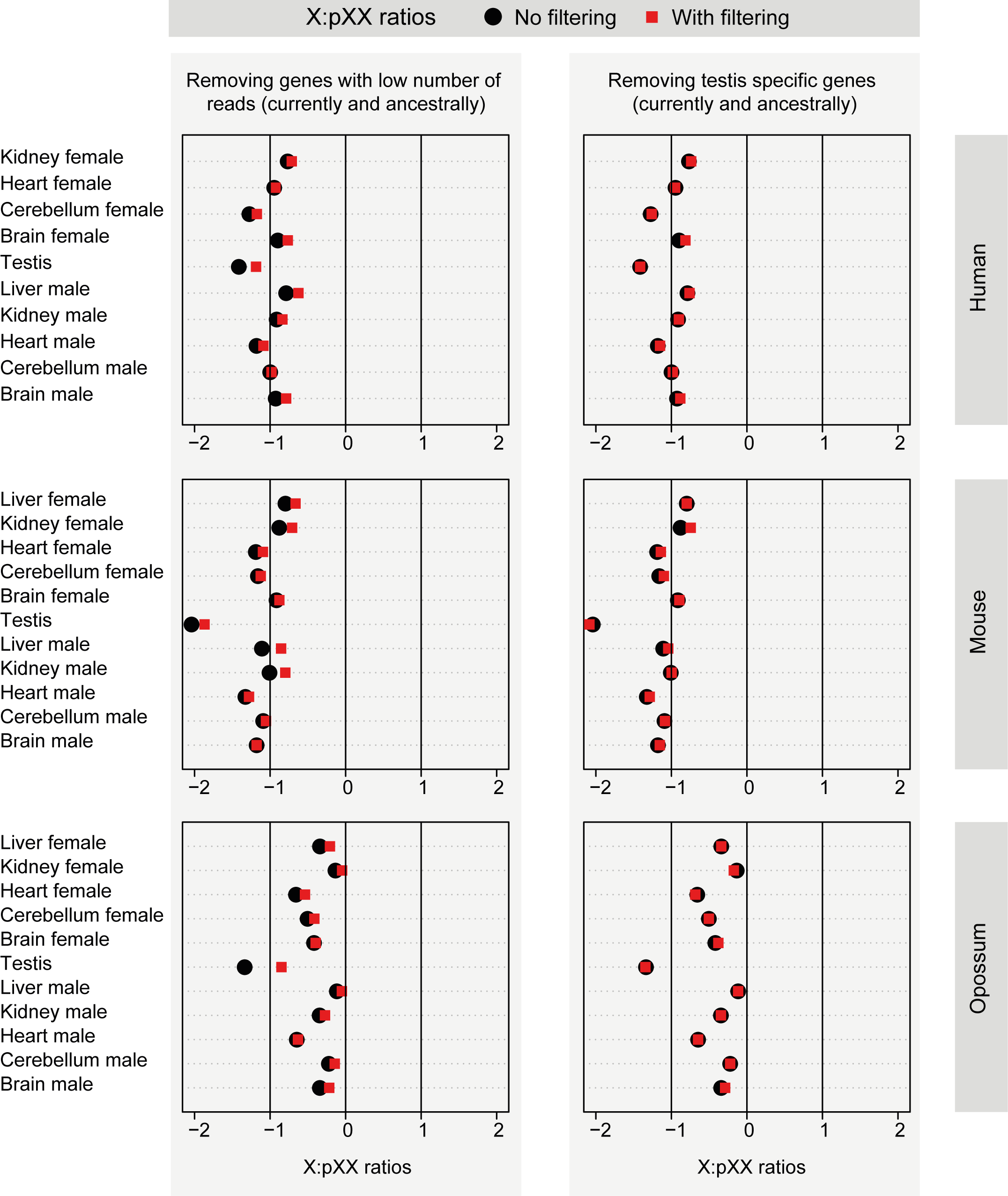

Supplement: Figure S7 — X to proto-X expression ratios for different gene sets (human, mouse, opossum). Left: X∶pXX ratios calculated for genes with ≥1 read (black circles) or ≥3 reads (red squares) on both the current X and proto-X. Right: X∶pXX ratios for all expressed genes (black circle) or all genes except testis specific genes (red squares). Values are plotted on a log2 scale (e.g., 0.5 [log2 ratio of −1]; 1 [log2 ratio of 0]; and 2 [log2 ratio of 1]). See Table S2 for values in all species and tissues. (TIF) [file pbio.1001328.s007.tif]

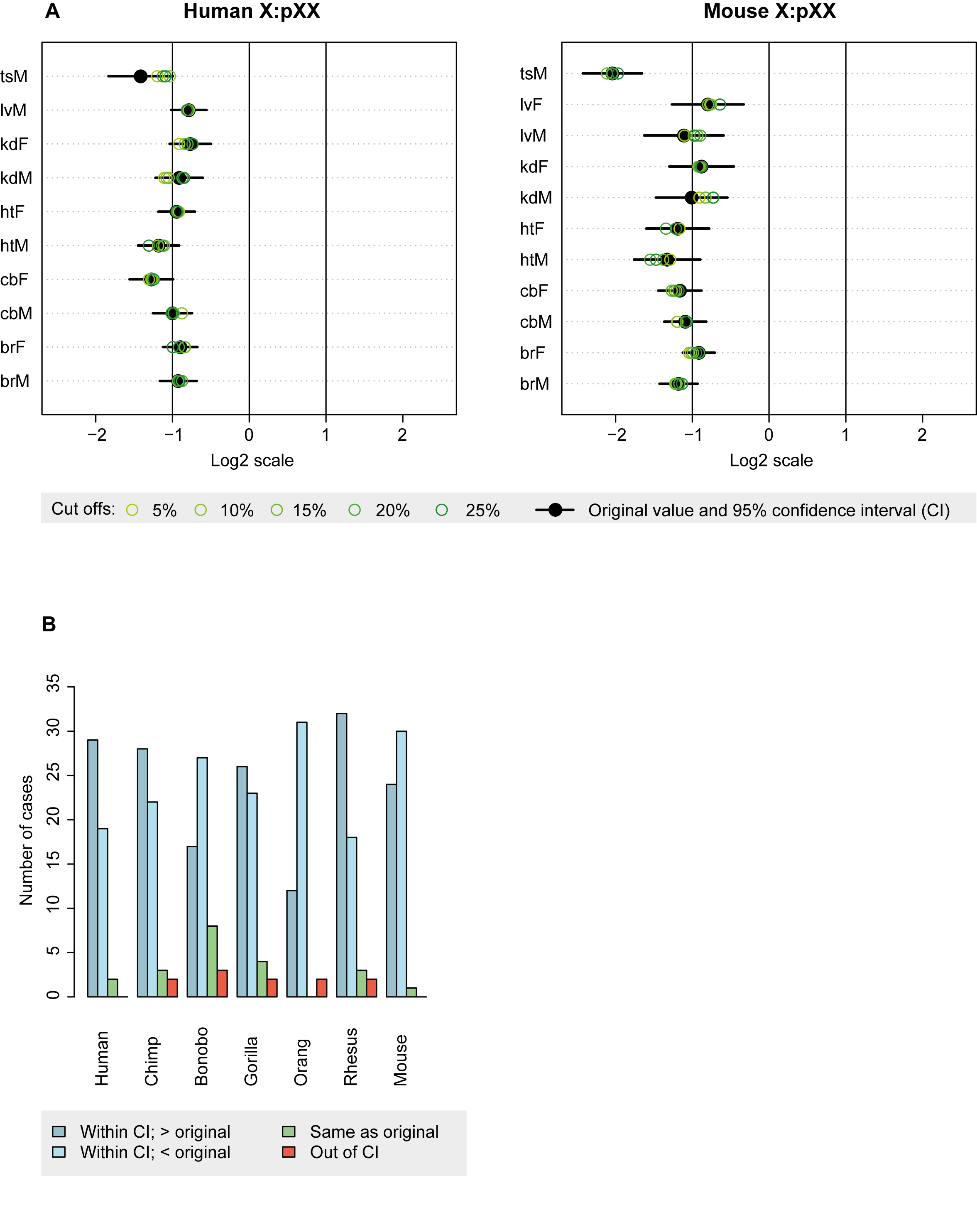

Supplement: Figure S8 — X to proto-X expression ratios when excluding various proportions of genes with lower expression levels. (A) Original X∶pXX ratios (black circles) and 95% confidence intervals (black lines) for all expressed genes are shown. Green circles indicate X∶pXX values calculated for datasets where various proportions of the most lowly transcribed genes for both the current X and proto-X are excluded (see Text S1 for details). Sample abbreviations: br, brain; cb, cerebellum; ht, heart; kd, kidney; lv, liver; ts, testis; M, male; F, female. (B) Comparisons of X∶pXX values based on trimmed and untrimmed data. For each eutherian species studied, the number of times the computed X∶pXX ratios fall within or outside the original 95% confidence intervals are shown. (TIF) [file pbio.1001328.s008.tif]

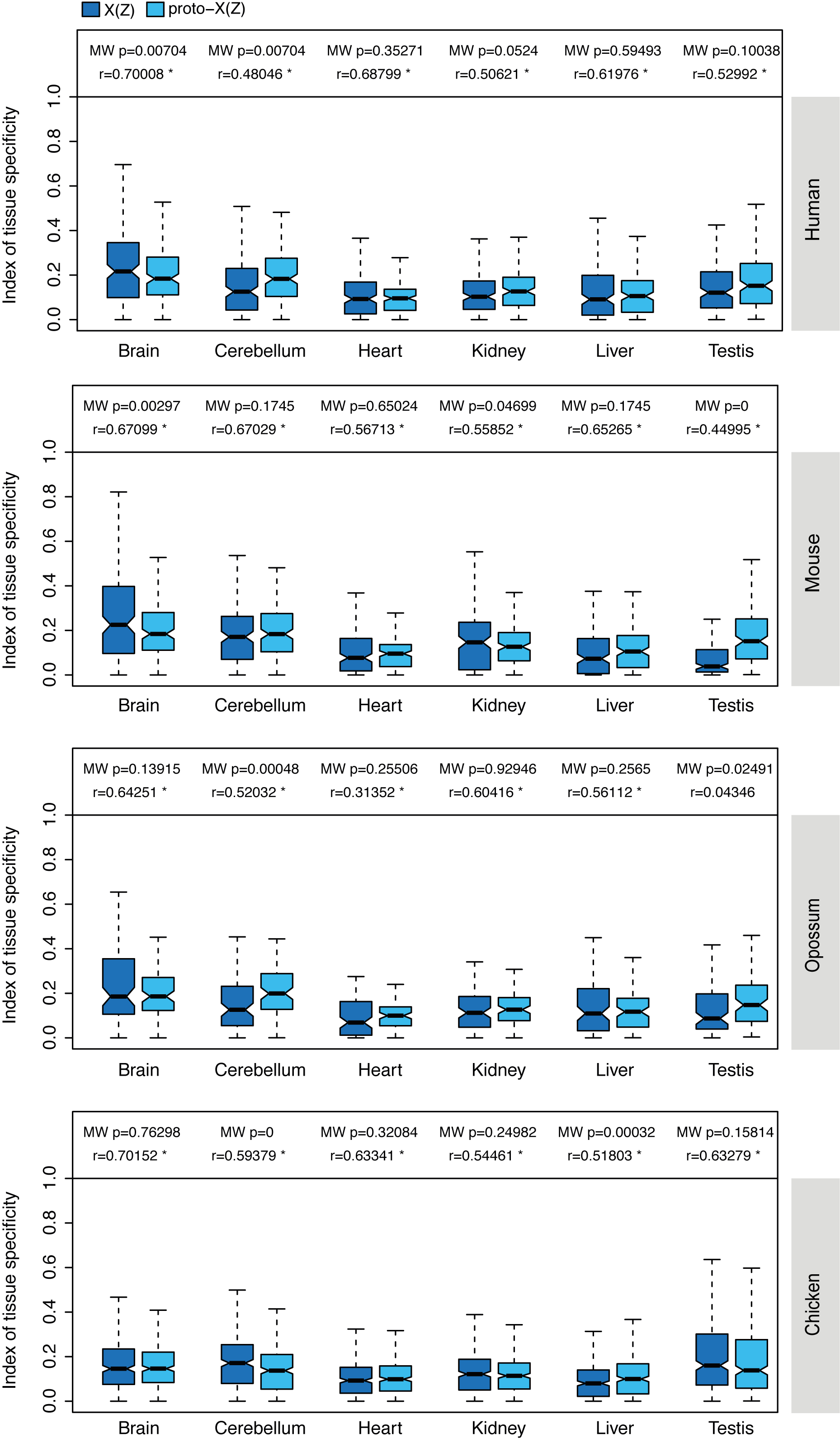

Supplement: Figure S9 — Tissue-specificities of genes on the X (Z) and proto-X (Z) chromosomes in amniotes. Distributions of the individual tissue specificity indices for the X (Z) and proto-X (Z) chromosomes are plotted. Statistical differences between current and proto sex chromosomes, as assessed by paired Mann-Whitney U tests (MW), are indicated. Also, the correlations between tissue-specificity indices of current and ancestral genes are indicated (correlation coefficient, r). An asterisk indicates statistically significant correlations (p<0.05). (TIF) [file pbio.1001328.s009.tif]

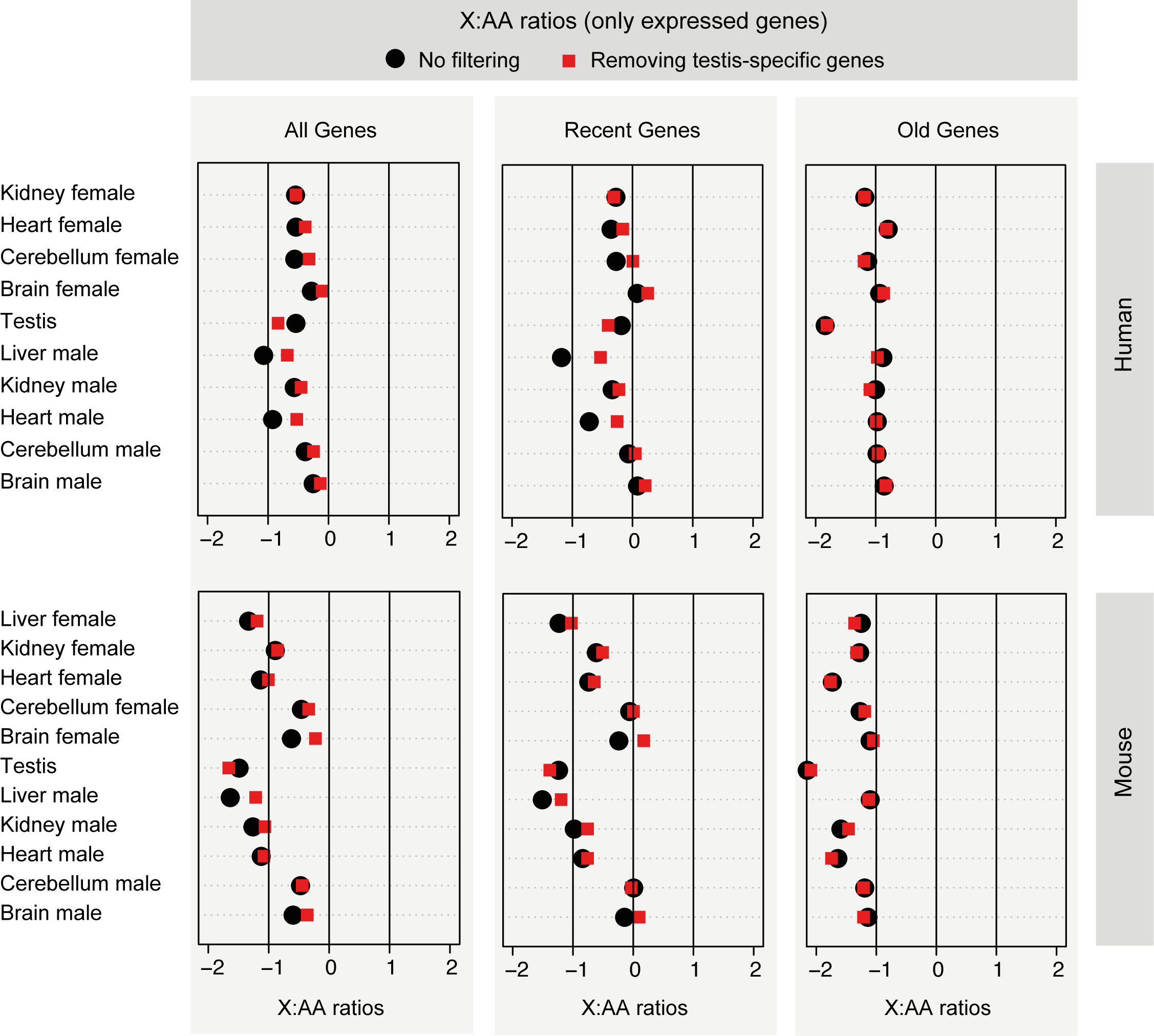

Supplement: Figure S10 — Human and mouse X∶AA ratios for recent and old genes (human, mouse, and opossum). X∶AA ratios calculated for expressed genes (black circles) or expressed genes except testis-specific genes (red squares) for three different sets of X-linked genes: All expressed genes (left), a set of genes enriched for genes that accumulated since sex chromosome differentiation (“Recent Genes,” middle panels), and 1∶1 orthologs present on the current X and proto-X (“Old” genes, right). See Table S2 for values in all species and tissues. (TIF) [file pbio.1001328.s010.tif]

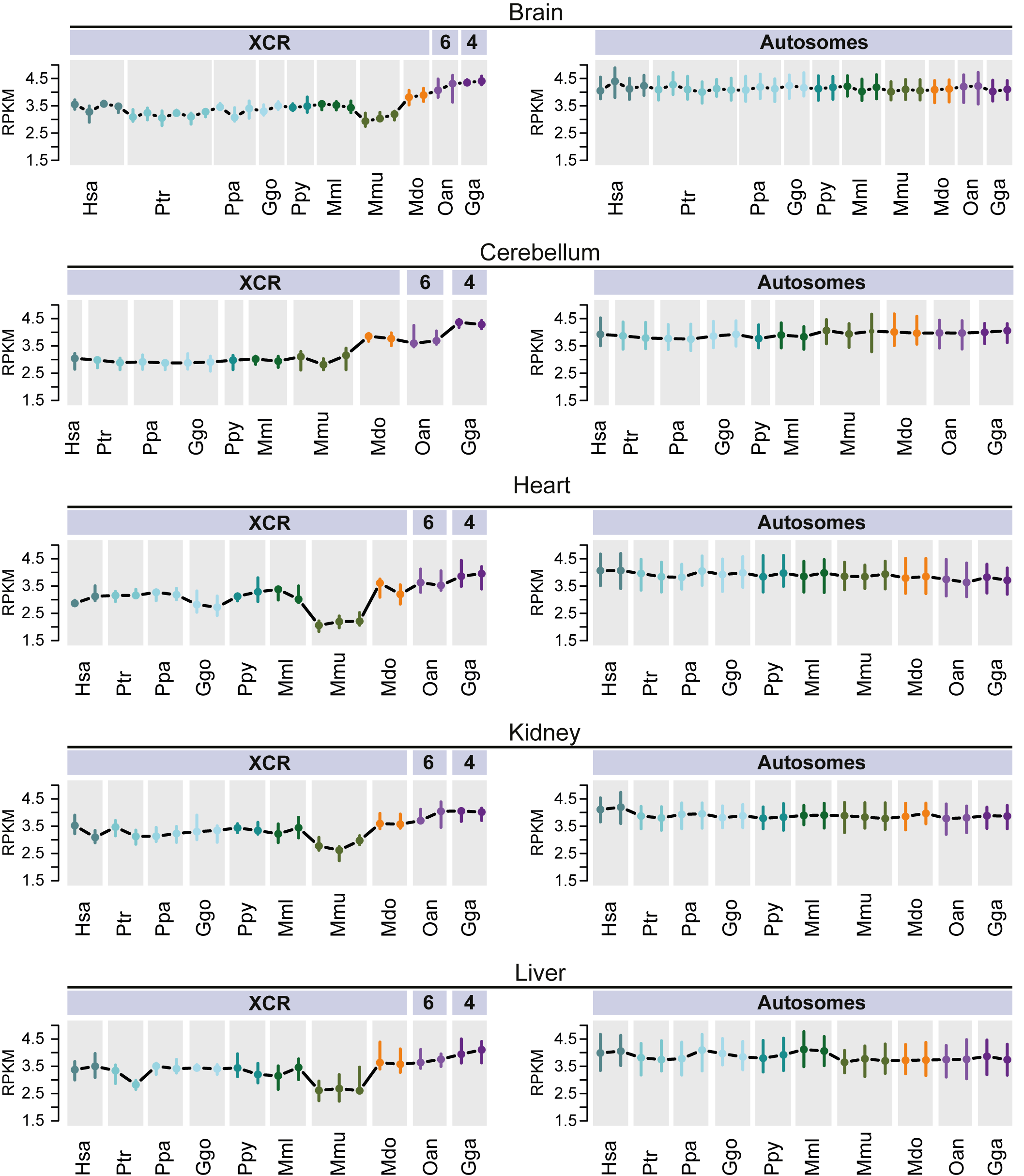

Supplement: Figure S11 — Expression levels of therian genes in the X-conserved region and their autosomal counterparts in platypus and chicken. Left: global expression levels (based on third quartiles of the RPKM distribution) of genes in the therian XCR (see Figure 3B for details) and their autosomal orthologs in outgroup species with different sex chromosome systems (see Figure S7 for all five somatic tissues). Error bars represent the range containing 90% of the third quartiles of individual resampling sets (80% of 90 orthologous genes were resampled 100 times). Right: expression levels of resampled sets of 90 genes that are autosomal in all ten species. The central value is the median of the third quartiles of resampled sets (error bars represent the central 90% of the distribution of those third quartiles). Has, human; Ptr, chimp; Ppa, bonobo; Ggo, gorilla; Ppy, orang; Mml, macaque; Mmu, mouse; Mdo, opossum; Oan, platypus; Gga, chicken. (TIF) [file pbio.1001328.s011.tif]

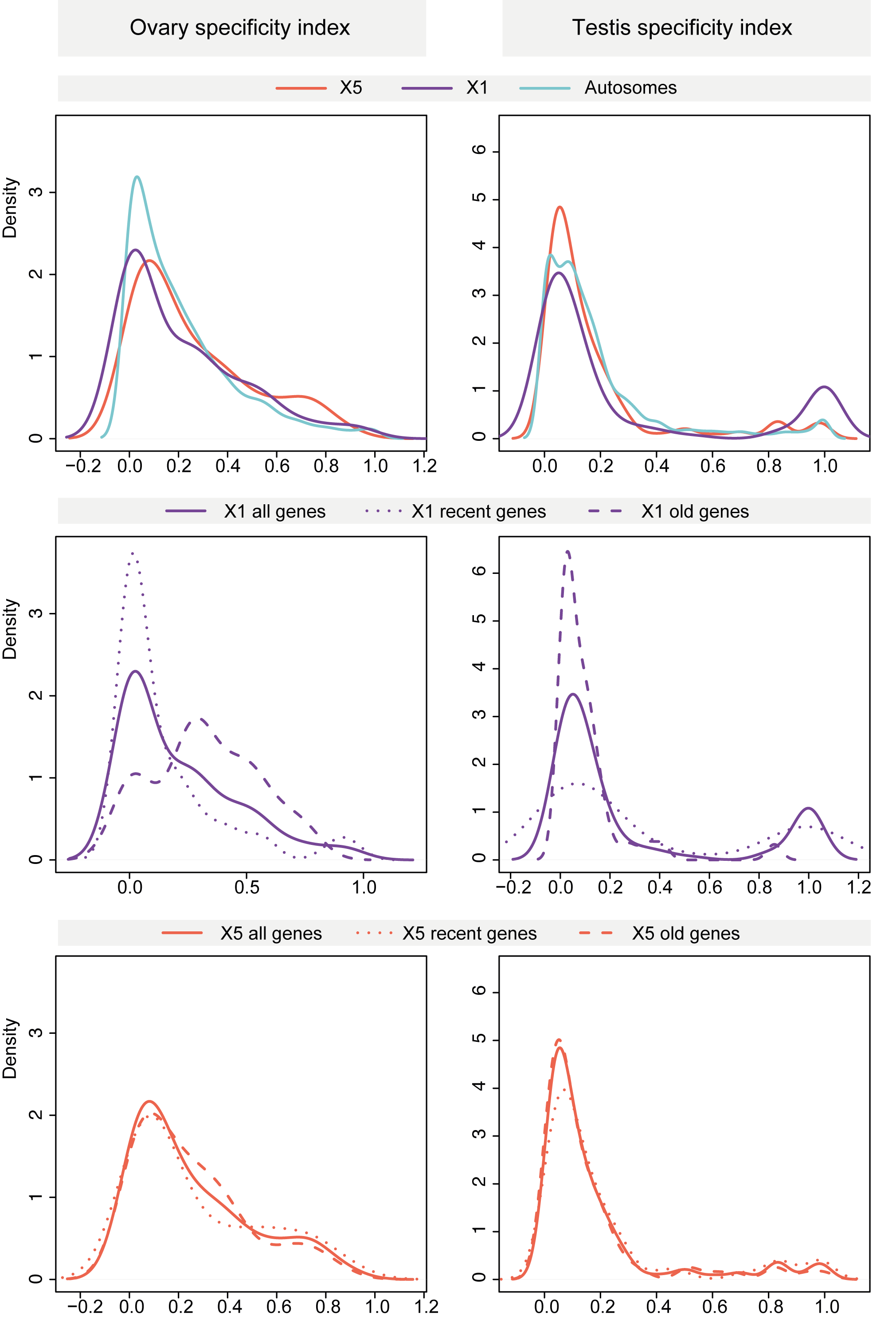

Supplement: Figure S12 — Patterns of testis and ovary specificities on the platypus X1 and X5 chromosomes. Density plots of ovary and testis specificity indices (Methods) for genes on the platypus X1 and X5 chromosomes. “Recent genes”: all genes except 1∶1 orthologs present on both the current X and proto-X chromosomes; “Old genes”: 1∶1 orthologs present on both the current X and proto-X chromosomes. (TIF) [file pbio.1001328.s012.tif]

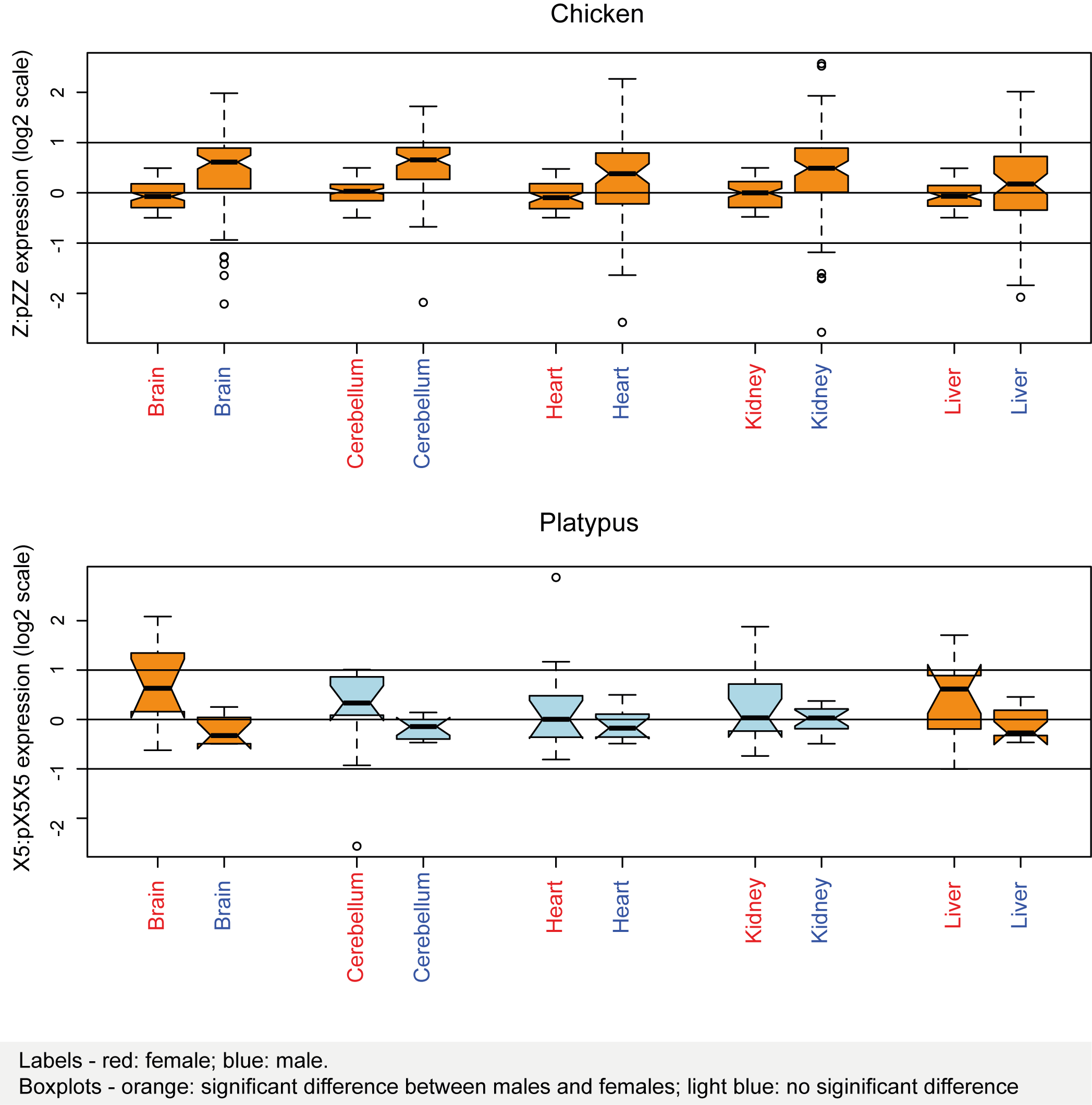

Supplement: Figure S13 — Sex chromosomal genes strongly upregulated in the heterogametic sex of platypus and chicken. For each tissue, we defined subsets of X(Z)-linked genes for which current and ancestral expression levels (see Methods for details regarding their calculation) were very similar in the heterogametic sex of chicken and platypus (i.e., log2 X∶pXX or Z∶pZZ ratio between 0.71 and 1.41 [−0.5 and 0.5 in a log2 scale, respectively]; note that using more stringent log2 thresholds of 0.81 and 1.23 [−0.3 and 0.3 in a log2 scale, respectively] gave similar results)]. We then assessed the distribution of the extent of upregulation for these subsets of genes in the homogametic sex. Values are plotted on a log2 scale to allow for linear and symmetrical patterns. (TIF) [file pbio.1001328.s013.tif]

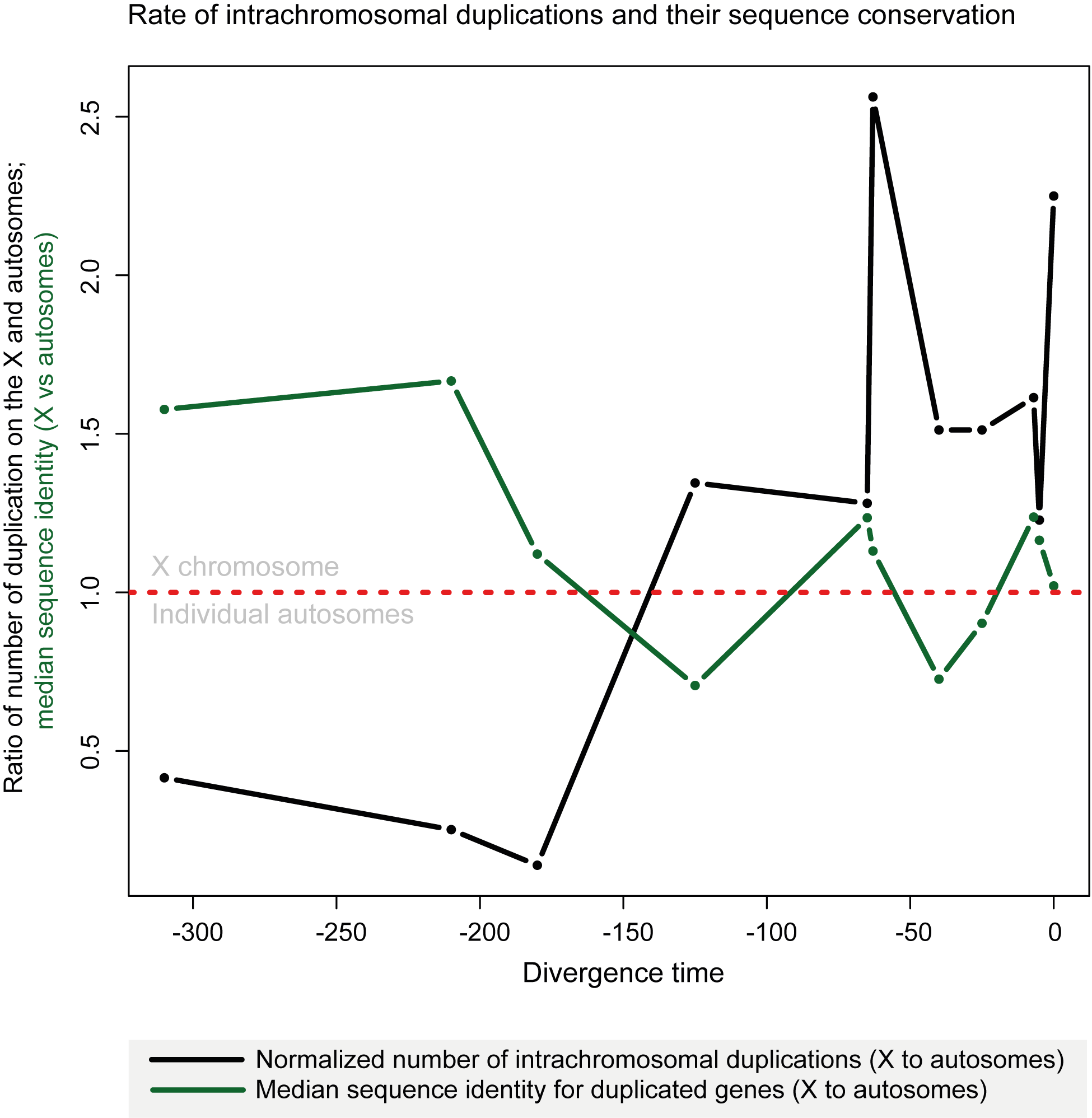

Supplement: Figure S14 — Rates of gene duplication on the X and autosomes during amniote evolution and duplicate gene preservation. The rate of intra-chromosomal protein-coding gene duplication is indicated in black; sequence identity of proteins encoded by the corresponding duplicated genes is indicated in green. See Methods for details on identification and dating of duplication events as well as sequence conservation analysis. (TIF) [file pbio.1001328.s014.tif]

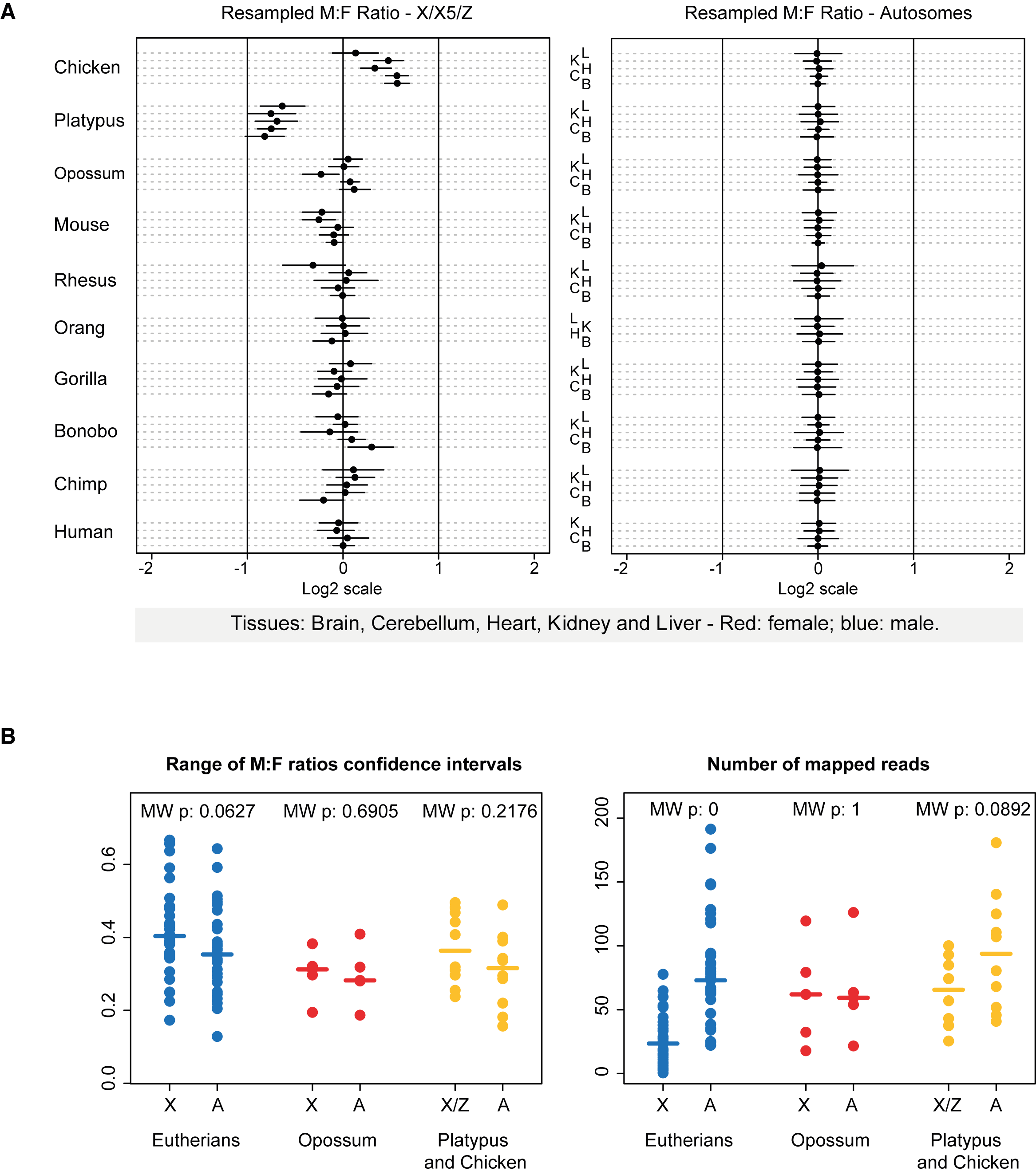

Supplement: Figure S15 — Gene expression variance on sex chromosomes and autosomes. (A) Resampling analysis of the male to female (M∶F) expression ratio of sex chromosome-linked and autosomal genes in the ten different species. For both sex chromosome-linked genes and autosomal genes, we resampled (100 times) 80% of the total number of sex chromosome-linked genes for each species and tissue (medians of the upper and lower bounds of the 95% confidence intervals of each resampling set are indicated). Note that values are plotted on a log2 scale to allow for linear and symmetrical patterns. (B) Left: distribution of the range of confidence intervals indicated above for sex chromosomes and autosomes, respectively. Right: distribution of the median number of mapped RNA-seq reads for the same sets of sex chromosome-linked and autosomal resampled genes. Statistical differences (p-values, p) between the respective distributions, as assessed by Mann-Whitney U tests (MW), are indicated. (TIF) [file pbio.1001328.s015.tif]

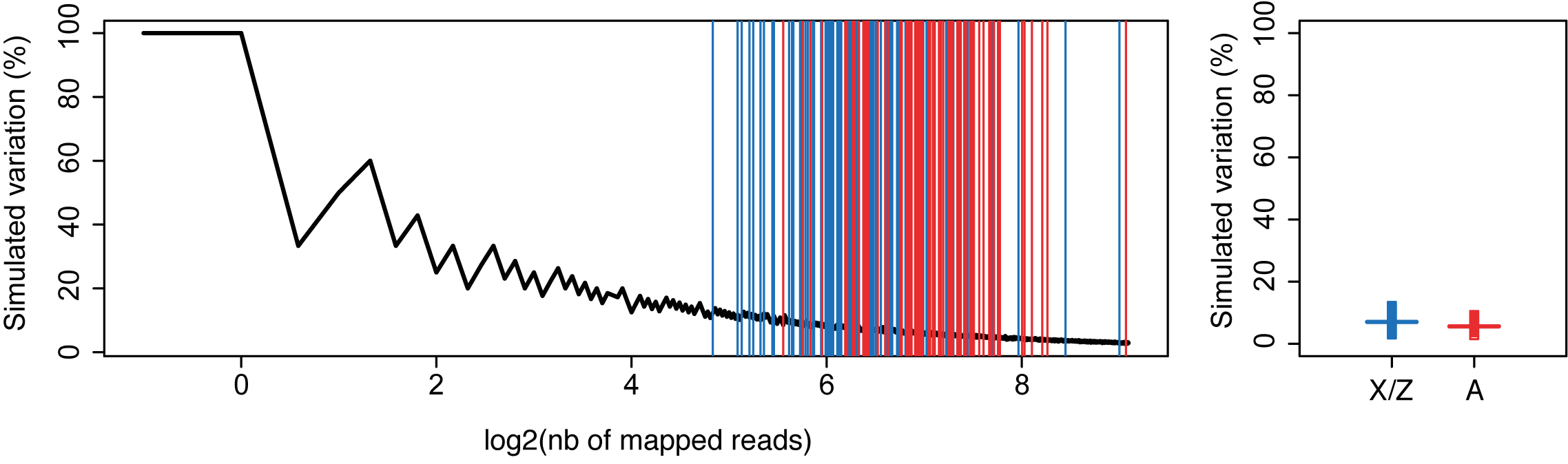

Supplement: Figure S16 — Expected random variation of median expression levels in function of number of mapped reads. Left: The plot shows estimated read sampling variations given theoretical numbers of mapped reads (black curve). Actual median number of reads observed for X-linked (blue lines) and autosomal genes (red lines) in our different biological samples are indicated. See Methods for details of the simulations procedure. Right: Range and median of expected percentage of random variation for the actual median number of reads observed for X-linked and autosomal genes in our different biological samples. (TIF) [file pbio.1001328.s016.tif]

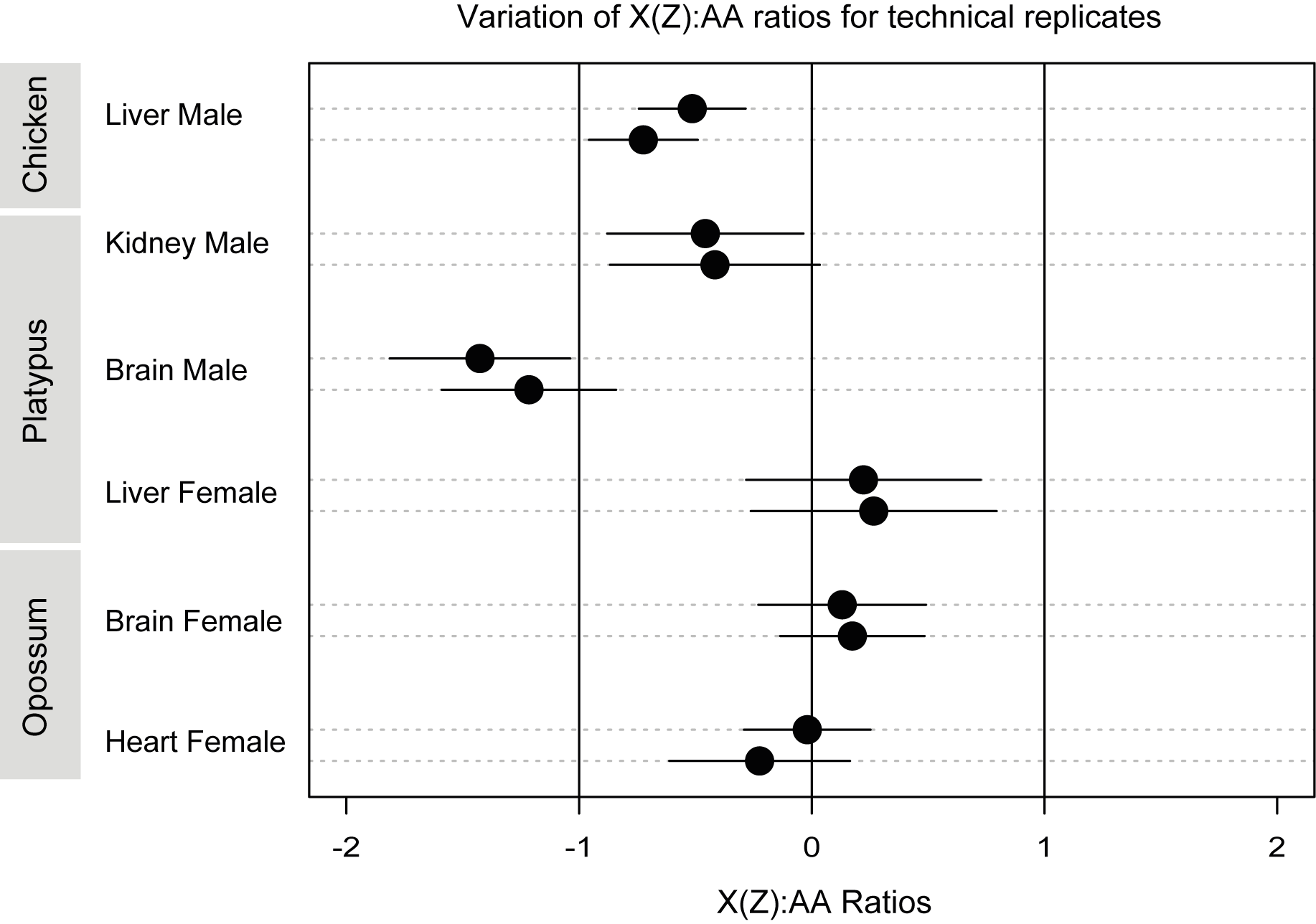

Supplement: Figure S17 — Variation of X∶AA estimates among technical replicate RNA-seq datasets. Median X (Z) to autosome expression level ratios and 95% confidence intervals of all expressed genes for six sets of replicate RNA-seq data are shown. The respective sets represent separate lanes of different Illumina GA IIx runs for the same RNA-seq library. The ratios in the respective pairwise comparisons are not significantly different from each other (p>0.15, Mann-Whitney U test). The number of reads for each lane ranges from 1.7–33 million reads. Notably, the respective datasets for each species' tissue were pooled for the main analyses represented in the paper. (TIF) [file pbio.1001328.s017.tif]

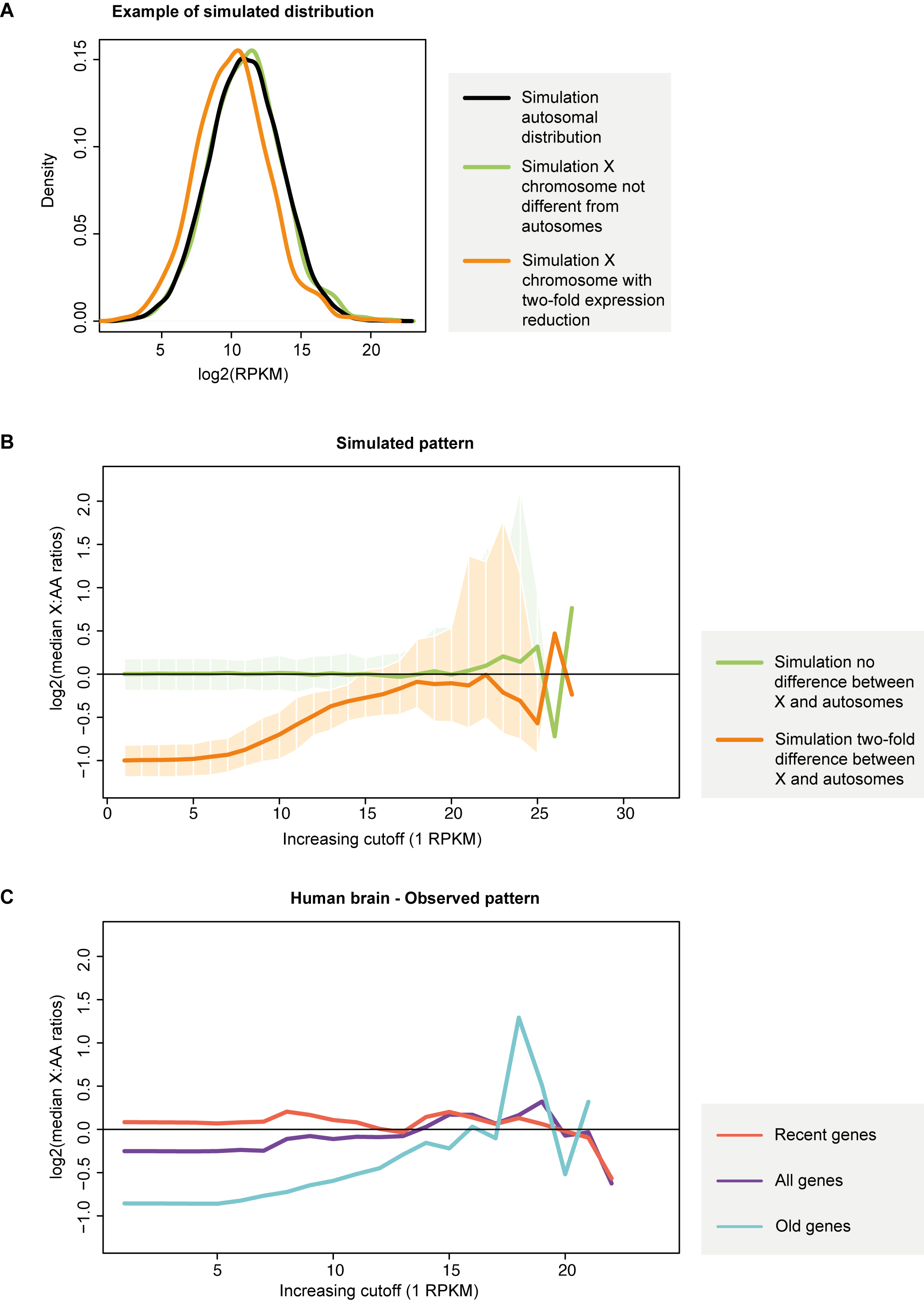

Supplement: Figure S18 — Effect of increasing expression level thresholds for the calculation of X∶AA ratios. (A) Effect of increasing expression level thresholds on observed X∶AA ratios for human brain. Separate curves are shown for all expressed genes, recent genes (i.e., all genes except 1∶1 orthologs present on both the current chromosomes and ancestral/proto chromosomes), and old genes (i.e., 1∶1 orthologs present on both the current chromosomes and ancestral/proto chromosomes), respectively. (B) Black curve: random simulation of expression level distribution for autosomal genes, which is based on observed median, standard deviation, and number of expressed autosomal genes in human brain (AA set). Green curve: random simulation of expression level distribution for X-linked genes based on observed median and standard deviation of expressed autosomal genes in human brain, and the number of expressed X-linked genes in human brain (X1 set). This distribution thus reflects a set of X-linked genes whose overall distribution is not different from autosomal genes. Orange curve: same distribution as the one represented by the green curve, but with a two-fold reduction of expression levels for each gene (X2 set). (C) Effect of increasing expression level thresholds on simulated X∶AA ratios based on simulated expression level distributions such as the ones described in (B). Green curve: the curve represents the median X1∶AA ratio for 1,000 generated random distributions of X1 and AA expression levels. Orange curve: The curve represents the median X2∶AA ratio for 1,000 generated random distributions of X2 and AA expression levels. The range covering 90% of the computed ratios is shown by the respective shadings. Together, these results show that gradually increasing expression level thresholds will gradually increase X∶AA ratios and eventually lead to X∶AA ratios around 1 (log2 ratio of 0), in spite of actual two-fold lower expression levels of X-linked genes. (TIF) [file pbio.1001328.s018.tif]
