# Supplementary material for: Mechanisms and Evolutionary Patterns of Mammalian and Avian Dosage Compensation
Source: PLoS Biol. 2012 May 15;10(5):e1001328. doi: 10.1371/journal.pbio.1001328 (PMC3352821; doi:10.1371/journal.pbio.1001328)
Supplement: Text S1 — Discussion of expression cutoffs. (DOC) [file pbio.1001328.s022.doc]

**Supplementary Text**

Comparisons of X and autosomal expression levels in our study are based on expressed genes (RPKM > 0), consistent with previous suggestions . It was further suggested that a more stringent criterion than the detection of at least one associated RNA-seq read should be used for the robust identification of transcriptionally active genes, given uncertainties regarding read mapping . Although we used a very stringent read mapping procedure (see ref. for details), we cannot exclude the occurrence of individual read mapping ambiguities. Thus, we also performed X:pXX (Z:pZZ) ratio calculations for expressed genes with the requirement that at least 3 reads map to them on both the current X (Z) and inferred proto-X (Z) chromosomes (i.e., on the autosomal 1:1 orthologous gene in outgroup species with different sex chromosome systems). This analysis shows that ratios calculated with this criterion are very similar to the ratios obtained when requiring only ≥1 read for defining expressed genes (Figure S7 and Table S2). Thus, our results are robust to modifications in the definitions of transcriptionally active genes.

Deng et al. suggested that the overrepresentation of testis-specific genes on the X may confound analysis of X upregulation, given that these genes may have no function and hence low expression levels in somatic tissues . This issue is extensively treated in our analyses that are presented in the main text.

In addition, these authors more generally noted that genes with low (lower) expression levels may confound expression level comparisons between the X and autosomes and then show that X:AA expression ratios increase with increasing expression level cutoffs. However, we note that such an analysis is problematic due to circular reasoning. Specifically, if we assume that the X has indeed not been (completely) upregulated after sex chromosome differentiation, then genes on the X have overall reduced expression levels compared to genes on autosomes. If genes with lower expression levels, which are thus overrepresented on the X, are then gradually removed, X and autosomal gene expression levels will be forced to become more and more similar. This is because the set of genes that remain on both chromosomes are gradually restricted to genes from higher expression classes, which are more similar between the two types of chromosomes and still exist on both the X and autosomes. To illustrate this, we generated simulated distributions of X-linked and autosomal genes, where X-linked genes have precisely a two-fold reduction of expression levels compared to autosomal genes (Figure S18A). We then calculated X:AA expression ratios for genes, gradually restricting the analysis to genes with higher minimum expression levels. This reveals that X:AA expression ratios become more similar with increasing expression level cutoffs, eventually leading to X:AA ratios of 1, in spite of the actual twofold reduced expression levels (Figure S18B). Notably, the simulated pattern is very similar to that observed using real data (Figure S18C). Thus, while it is important to base expression level comparisons of the X and autosomes on expressed genes and also to consider patterns of tissue/testis-specificity in such analyses, further trimming of the data based on increasing expression level thresholds will lead to artificially inflated X:AA estimates.

However, for the X:pXX calculations, the data can be further trimmed (i.e., in addition to the restrictions associated with the definition of transcribed genes; see above), given that expression levels of the “same” genes are compared in this analysis (i.e., X-linked genes and their autosomal orthologs from outgroup species; see main text). Thus, to rule out that our X:pXX analyses are confounded by genes with low expression levels, we removed between 5% to 25% of the most lowly transcribed genes for both the current X and proto-X, and then calculated X:pXX values for the remaining common (orthologous) genes. Notably, the X:pXX values resulting from this analysis are very similar to those of the original analysis (see Figure S8A for human and mouse results, shown as examples). When jointly considering all eutherians and all different expression level cutoffs, only 2.97% (11 cases out of 370) of the X:pXX values fall outside of the 95% confidence intervals of the originally computed X:pXX values (Figure S8B). Furthermore, among the X:pXX values falling within the original 95% confidence intervals, 168 are greater than the original X:pXX values, while 170 are smaller (the remaining 21 X:pXX values are identical to the original value). These observations show that the removal of various proportions of genes with lower expression levels leads to X:pXX values that are very similar to the original ones where only non-expressed genes were removed (i.e., they randomly fluctuate around the original value). Thus, the X:pXX analyses presented in the main text, central to our conclusions, are not confounded by lowly transcribed genes.

**References**

1. Deng X, Hiatt JB, Nguyen DK, Ercan S, Sturgill D, et al. (2011) Evidence for compensatory upregulation of expressed X-linked genes in mammals, Caenorhabditis elegans and Drosophila melanogaster. Nat Genet.

2. Kharchenko PV, Xi R, Park PJ (2011) Evidence for dosage compensation between the X chromosome and autosomes in mammals. Nat Genet 43: 1167-1169.

3. Brawand D, Soumillon M, Necsulea A, Julien P, Csárdi G, et al. (2011) The evolution of gene expression levels in mammalian organs. Nature 478: 343-348.
